# Detection of *Mycobacterium tuberculosis* Peptides in the Exosomes of Patients with Active and Latent *M. tuberculosis* Infection Using MRM-MS

**DOI:** 10.1371/journal.pone.0103811

**Published:** 2014-07-31

**Authors:** Nicole A. Kruh-Garcia, Lisa M. Wolfe, Lelia H. Chaisson, William O. Worodria, Payam Nahid, Jeff S. Schorey, J. Lucian Davis, Karen M. Dobos

**Affiliations:** 1 Department of Microbiology, Immunology and Pathology, Colorado State University, Fort Collins, Colorado, United States of America; 2 Proteomics and Metabolomics Facility, Colorado State University, Fort Collins, Colorado, United States of America; 3 Division of Pulmonary and Critical Care Medicine, San Francisco General Hospital, University of California San Francisco, San Francisco, California, United States of America; 4 Department of Medicine, Mulago Hospital, Makerere University, Kampala, Uganda; 5 Curry International Tuberculosis Center, San Francisco General Hospital, University of California San Francisco, San Francisco, California, United States of America; 6 Department of Biological Sciences, Eck Institute for Global Health, University of Notre Dame, Notre Dame, Indiana, United States of America; Moffitt Cancer Center, United States of America

## Abstract

The identification of easily measured, accurate diagnostic biomarkers for active tuberculosis (TB) will have a significant impact on global TB control efforts. Because of the host and pathogen complexities involved in TB pathogenesis, identifying a single biomarker that is adequately sensitive and specific continues to be a major hurdle. Our previous studies in models of TB demonstrated that exosomes, such as those released from infected macrophages, contain mycobacterial products, including many *Mtb* proteins. In this report, we describe the development of targeted proteomics assays employing multiplexed multiple reaction monitoring mass spectrometry (MRM-MS) in order to allow us to follow those proteins previously identified by western blot or shotgun mass spectrometry, and enhance biomarker discovery to include detection of *Mtb* proteins in human serum exosomes. Targeted MRM-MS assays were applied to exosomes isolated from human serum samples obtained from culture-confirmed active TB patients to detect 76 peptides representing 33 unique *Mtb* proteins. Our studies revealed the first identification of bacteria-derived biomarker candidates of active TB in exosomes from human serum. Twenty of the 33 proteins targeted for detection were found in the exosomes of TB patients, and included multiple peptides from 8 proteins (Antigen 85B, Antigen 85C, Apa, BfrB, GlcB, HspX, KatG, and Mpt64). Interestingly, all of these proteins are known mycobacterial adhesins and/or proteins that contribute to the intracellular survival of *Mtb*. These proteins will be included as target analytes in future validation studies as they may serve as markers for persistent active and latent *Mtb* infection. In summary, this work is the first step in identifying a unique and specific panel of *Mtb* peptide biomarkers encapsulated in exosomes and reveals complex biomarker patterns across a spectrum of TB disease states.

## Introduction

The current standard for diagnosis of TB in high burden settings is sputum acid-fast bacilli smear microscopy. While this method identifies individuals with active disease at highest risk of transmitting *Mtb*, it has a sensitivity as low as 50% [1]. This problem is intensified in patients who are unable to produce sputum such as children and human immunodeficiency virus (HIV) infected patients, two groups who are highly susceptible to TB and who suffer high morbidity and mortality from TB [2]–[4]. An inability to accurately diagnose disease can result in delayed treatment while prolonging the window of opportunity for TB transmission to household contacts and the community [5]. An alternative diagnostic approach to sputum smear microscopy is needed, and ideally would avoid sputum entirely and instead use blood, which can be readily collected. Unfortunately, existing serodiagnostics perform so poorly that the World Health Organization has taken the exceptional step of issuing a negative diagnostic recommendation [6]. Therefore, new approaches are needed to harness the diagnostic capacity of serum.

The identification of a bacteria-specific biomarker in serum poses a unique challenge, namely the complexity of the host protein content of this biological fluid, making the detection of bacteria-specific proteins difficult. Serum is composed of over ten thousand proteins that vary in abundance across at least nine orders of magnitude, ranging from milligram/milliliter (albumin) to picogram/milliliter (interleukins) [7], [8]. Bacterial components are hypothesized to be near the lower limit of detection. To overcome this hurdle, the vesicle population, specifically exosomes, was purified from whole serum greatly enriching for the bacterial proteins we postulate to be present within these vesicles (exo-proteome). Exosomes are 30–100 nm membrane-bound vesicles constitutively released from most eukaryotic cell types into the lymphatic system and blood to facilitate systemic, as well as local intercellular communication. Exosomes are an ideal source of biomarkers for other chronic diseases, since their release appears to be stimulated by inflammation and hypoxia [9] and their contents are attributed to the cell of origin, reflecting cellular abnormalities and disease state [10]. We hypothesize that the majority of mycobacterial proteins, at least in serum, are localized to exosomes. This is based on the fact that *Mtb* is an intracellular pathogen which is known to release mycobacterial components from the phagosome during infection [11]. While the mechanism by which mycobacterial components are trafficked from the multivesicular body into exosomes is unknown, the purification of exosomes from serum would be expected to greatly enrich for mycobacterial components including proteins and thus be a good source for biomarker identification. In support of this hypothesis, we recently identified over 250 potential biomarkers, both host and pathogen derived, from exosomes purified from *Mtb*-infected macrophages and biological fluids from *Mtb*-infected animal models [12], [13]. Despite being less abundant, bacterial biomarkers have the theoretical advantage of increased specificity, which we hypothesize will provide distinct peptide fingerprints indicative of active TB.

Here, we report the first evidence of *Mtb* proteins found in exosomes isolated from human serum and the successful generation of a proteomic workflow for bolstering the number of bacterial peptides identified from a simplified fraction of blood. The MRM platform allows for the rational development of multiplexed assays for increasing our capacity to detect several novel *Mtb* biomarkers in serum. Biomarkers detected from these assays will be the subject of future validation studies and ultimately, the development of novel peptide bioassays diagnostic of active TB.

## Materials and Methods

### Ethics Statement

The study was approved by the UCSF Committee on Human Research (#10-02633), the Makerere University School of Medicine Research Ethics Committee (#2006-017), the Mulago Hospital Research and Ethics Committee (Reference MIND Study), and the Uganda National Council for Science and Technology (#HS 259); all participants consented to participation via written consent. All animals were handled in strict accordance with good animal practice as defined by the relevant national and/or local animal welfare bodies, and all animal work was approved by the Colorado State University Institutional Animal Care and Use Committee (IACUC #10-2306A).

### Study Participants

Between July 2007 and July 2009, two groups of patients were prospectively enrolled; both groups were newly admitted to the medical wards of Mulago Hospital (Kampala, Uganda) with cough ≥2 weeks. One group (“discovery group” n = 8) was a randomly selected case series of patients with confirmed culture-positive pulmonary TB, and the second group (“qualification group” n = 59) was a consecutive random sample of adults (age ≥18 years). Samples were collected from TB cases and non-TB controls nested within our cohort of consecutive patients with chronic unexplained cough, using pre-established reference standard criteria (Methods S1; Figure S1; Table S1). Patients with concurrent or previous TB treatment in the previous two years were excluded. Demographic and clinical information from all patients was collected using a standardized questionnaire. All patients underwent chest radiography, collection of blood for HIV antibody testing, CD4+ T-lymphocyte counts, and T-cell interferon gamma release assay testing (T.SPOT.TB, Oxford Immunotec, Oxford, UK), and collection of sputum samples over two consecutive days for acid-fast bacilli smear microscopy and culture, as part of a previously described comprehensive clinical evaluation [14]. Technicians at the Uganda National TB Reference Laboratory performed all mycobacterial studies according to standard protocols [15]. Those performing laboratory assays were initially blinded to all clinical characteristics and disease classification.

### Sample Preparation

Laboratory investigators (NKG, LMW, JSS, and KMD) were blinded to patient characteristics until completion of all assays and data generation. Exosomes were harvested from human serum via Exoquick (Systems Biosciences, Inc., Mountain View, CA) after filtration through a 0.2 micron filter. Protein content of the purified exosomes was quantitated by micro bicinchoninic acid assay (Thermo Fisher Scientific, Inc. Rockford, IL). Twenty µg of protein of each sample was resolved by SDS-PAGE and processed via digestion. Samples subjected to full proteomic characterization by LC-MS/MS were divided into ten gel fractions prior to digestion [12]. Exosome samples for MRM-MS were left unfractionated. All samples were digested with trypsin at a 20:1 ratio (sample:protease), as previously published [12].

### LC-MS/MS Discovery of Biomarker Candidates from Human Serum-Exosomes

Mass Spectrometry of tryptic peptides. One µL of each sample was injected at a concentration of approximately 500 ng/mL. Peptides were purified and concentrated using an on-line enrichment column (Agilent Zorbax C18, 5 mm, 560.3 mm column, Agilent 1100 nanoHPLC,). Subsequent chromatographic separation was performed on a reverse phase nanospray column (Zorbax C18, 5 mm, 75 mm ID 6 150 mm column). Samples were eluted into a LTQ linear ion trap (Thermo Scientific) using a flow rate of 300 nL/min with the following gradient profile: 0% B for 0–5 min, 0–15% B for 5–8 min, 15–55% B for 8–98 min, and 55–100% B for 98–103 min (A = 3% acetonitrile (ACN), 0.1% formic acid; B = 100% ACN, 0.1% formic acid). This elongated method has been optimized to separate complex samples, such as whole cell lysate. Mass spectra are collected over an m/z range of 200–2000 Da using a dynamic exclusion limit of 2 MS/MS spectra of a given mass for 30 s (exclusion duration of 90 s). Compound lists of the resulting spectra were generated using Bioworks 3.0 software (Thermo Scientific) with an intensity threshold of 5,000 and 1 scan/group.

### Database searching

All MS/MS samples were analyzed using Mascot (Matrix Science, London, UK; version Mascot), Sequest (Thermo Fisher Scientific, San Jose, CA, USA; version v.27, rev. 11), and X! Tandem (The GPM, thegpm.org; version 2007.01.01.1). Mascot was set up to search the TBv3_reverse_042110 database (042110, 181470 entries) assuming digestion with trypsin with a maximum of 1 missed cleavage; the searches were performed with a fragment ion mass tolerance of 1.50 Da and a parent ion tolerance of 2.5 Da. Sequest was set up to search the TBv3_human_rev_111510 database (111510, 186822 entries) assuming digestion with trypsin with a maximum of 2 missed cleavages; the searches were performed with a fragment ion mass tolerance of 1.0 Da and a parent ion tolerance of 1.5 Da. X! Tandem was set up to search the TB_ver3_reverse database (042110, 7980 entries) assuming digestion with trypsin with a maximum of 2 missed cleavages; the searches were performed with a fragment ion mass tolerance of 1.0 Da and a parent ion tolerance of 1.5 Da. Oxidation of methionine (+16) and iodoacetamide derivative of cysteine (+57) were specified as variable modifications. Multiple search engines were employed to improve the sensitivity and confidence of the peptides identified [16].

### Criteria for protein identification

Scaffold (version Scaffold 4.1.1, Proteome Software Inc., Portland, OR) was used to validate MS/MS based peptide and protein identifications. Peptide identifications were accepted if they could be established at greater than 90.0% probability by the Peptide Prophet algorithm [17]. Protein identifications were accepted if they could be established at greater than 90.0% probability and contained at least 1 identified peptide. All proteins identified were subject to manual validation. Protein probabilities were assigned by the Protein Prophet algorithm [18]. Proteins that contained similar peptides and could not be differentiated based on MS/MS analysis alone were grouped to satisfy the principles of parsimony. Scaffold output files are included as Document S1.

### Peptide Selection and Multiple Reaction Monitoring (MRM) Development

Skyline (64-bit) version 1.4.0.4421) was used to build and optimize the selected reaction monitoring (SRM) methods for the relative quantification of peptides [19]. FASTA-formatted sequences of all 36 proteins were used for *in* *silico* tryptic (KR|P) digestion and restricting the prediction of peptides to those ranging from 6 to 25 amino acids in length and assuming 100% digestion efficiency. Peptides which included cysteine and methionine were avoided when possible. The initial transitions selected for each peptide included both double and triple charged precursor ions, as well as, monitoring of both b and y ions. The resultant methods were exported to Masslynx (Waters Corporation, Milford, MA). Additional methods were developed on peptides identified in previous LC-MS/MS data sets. Skyline was then used to predict and optimize collision energies for each peptide [20]. The final methods included the selection of three transitions per peptide. All method development was initially performed on *Mtb* culture filtrate proteins, *Mtb* whole cell lysate, or recombinant protein if available (obtained by BEI Resources, NIH, NIAID, http://www.beiresources.org/). Final MRM methods were multiplexed as listed in Table S2 and included monitoring a total of 76 peptides by selecting for unique retention or dwell times ≥0.066 s whenever possible.

### Preparation of Matrix Controls for MRM Method Evaluation

Exosomes were isolated from the bronchoalveolar lavage (BAL) fluid, serum, and urine from experimentally *Mtb*-infected guinea pigs and uninfected controls. *Mtb*-infected guinea pigs were used as a positive control to confirm the presence of these peptides within one or multiple fluids. Guinea pigs were infected as previously published with modifications [21], [22]. Briefly, guinea pigs were infected with *Mtb* H37Rv by the low dose aerosol method (approximately 10^6^ colony forming units) with a Madison Chamber [22]. In addition, a small aliquot of the suspension was plated on 7H11 media to confirm dose of aerosolized bacilli used in each infection. Samples of urine and blood were collected after sedation with isoflurane, followed by catching spontaneously released urine into sterile, 5 ml polycarbonate snap-cap tubes and aseptic puncture of the subclavian vein. Blood was centrifuged at 10,000×g to remove cells and platelets and recover serum. Samples of BAL fluid were collected by instillation of ice-cold 2% heparin in phosphate-buffered saline (pH 7.4) into the lungs of euthanized guinea pigs [23]. All samples were filtered through a 0.2 µm filter prior to further use. Identical procedures were used for uninfected control guinea pigs. Exosomes from the serum of uninfected GPs were purified and used to determine the detection limits of select peptides across a range of concentrations by spiking synthetic peptides only, or peptides pre-mixed with trypsin-digested culture filtrate proteins or whole cell lysate into the exosome matrix. Poor peak intensity, low quality peak resolution, or inability to identify three transition ions in these control samples was used to eliminate candidate peptides from final assays. The raw spectral data is available for viewing at http://www.peptideatlas.org/PASS/PASS00329.

### Multiple Reaction Monitoring Mass Spectrometry

All analyses of method development and clinical samples were performed using seventeen 1 µL injections (concentrations ranging from 100 nM to 1 µg per µL) on a LC-MS/MS system consisting of a Waters nanoACQUITY UPLC coupled to a Waters TQ-S mass spectrometer fitted with a Trizaic source. The instrument was operated in positive electrospray ionization mode using MassLynx V4.1 SCN810 (Waters Corporation, Milford, MA). Chromatography was performed on an 85 µm×100 mm Trizaic nanotile packed with BEH C18 1.8 µm. Peptides were separated using gradient elution with a stable flow of 0.50 µL/min. Two gradients were utilized: Method 1 (24 minute run time) started with 76% buffer A (99.9% water with 0.1% formic acid) and 24% buffer B (99.9% ACN with 0.1% formic acid) for 3 minutes, followed by a 10 minute linear gradient to 32% B, and a 3 minute linear gradient to 85% B, followed by a 2 minute hold. The column was allowed to re-equilibrate at 12% B for 6 minutes prior to initiation of a second assay. Method 2 (15 minute run time) had an initial conditioning at 14% B, with a linear gradient of 7 minutes of 55% B. At 7.5 minutes, the column was flushed with 85% B for 2 minutes and then re-equilibrated down to 3% B for 5 minutes. The column was maintained at 46°C during analysis, and the samples were kept at 4°C. The MS was operating in selective reaction mode using electrospray ionization in positive ion mode, with a capillary voltage of 3.4 kV and a source temperature of 100°C. Cone voltage was static at 35 V and the collision energies were optimized for each compound individually (See Table S2). Peak identification and optimization was performed using either MassLynx software version 4.1 or Skyline.

### Spectral and Statistical Analysis

All spectral data was manually inspected in Skyline to confirm that auto-selected peak boundaries provided accurate integration and quantification. The raw spectral data is available for viewing at http://www.peptideatlas.org/PASS/PASS00330. Raw data from the MRM assays were processed and the sum of the peak areas for all monitored transitions was computed to provide the total peak area for each monitored peptide. Any peptide which did not contain a positive value for all three transition ions was not included in the analysis. From this data, the normal reference limit for each peptide was calculated by determining the maximum peak area in the TB- specimens [24], [25]. Boxplots were created to summarize the peak area data, enabling us to compare the TB+ and TB- samples, and ultimately, assist in establishing the normal reference limit for each target analyte. The normal reference limit is unique for each peptide and represents the lowest raw value in a positive sample that exceeded the highest value in a negative sample (excluding outliers or false positives). Peptides with a total peak area above the normal reference limit were considered positive (binary score = 1) and everything below negative (binary score = 0). The cumulative binary score across the 76 peptides was used to determine category averages. The results were evaluated using unpaired, two-tailed t-tests on the raw peak areas (GraphPad Prism, version 6.02 for Windows, GraphPad Software, La Jolla California USA, www.graphpad.com) to determine if the discriminatory peptides were statistically significant.

## Results

### Preliminary Biomarker Candidate Selection

**Figure 1:**
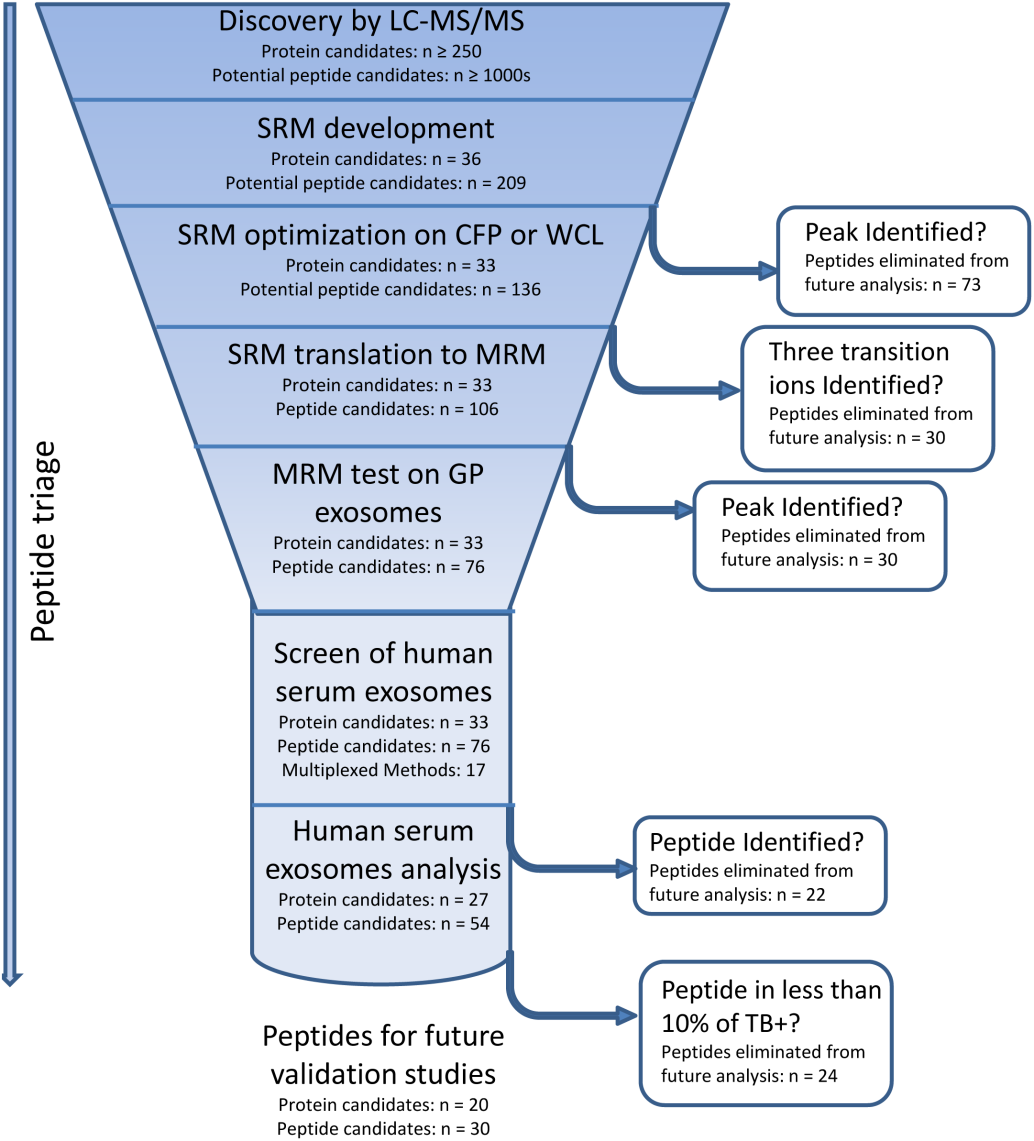
Flow diagram outlining the rational progression from the discovery phase of *Mtb* biomarkers using LC-MS/MS through the development of targeted MS assays for the identification of *Mtb* peptides in human biological fluids.

In designing the MRM assays we set out to confirm candidate protein biomarkers identified in three previous studies, including a screening of exosomes isolated from *Mtb*-infected macrophages, mice and humans. The comprehensive candidate list from these trials included over 250 proteins (Figure 1), however we chose to follow-up on: the most abundant proteins by spectral count; those which showed intriguing kinetic patterns in the mouse model; or those which were found in multiple systems. Specifically, 25 candidate proteins (of 33) were derived from our pilot discovery dataset in which mycobacterial proteins were identified by LC-MS/MS in exosomes isolated from *Mtb*-infected J774a.1 murine macrophage cells (Table 1) [12]; six of which, including the antigen 85 complex (Ag85), GroES, and CFP10, were also confirmed by western blot.

**Table 1:** Mycobacterial Proteins Included in the Final Seventeen MRM assays.

	Macrophage^1^	Mouse^2^	Human
Rv0009|ppiA			
Rv0066c|icd2		x	
Rv0125|pepA		x	
Rv0129c|Ag85c	x	x	
Rv0350|DnaK	x	x	x
Rv0363c|Fba		x	
Rv0440|groEL2		x	
Rv0934|PstS1	x	x	x
Rv1270c|lprA	x	x	
Rv1469|CtpD		x	x
Rv1827|GarA|Cfp17	x	x	
Rv1837c|GlcB	x	x	x
Rv1860|Apa|Mpt32	x	x	
Rv1886c|Ag85b	x	x	
Rv1908c|KatG	x	x	
Rv1926c|Mpt63	x	x	
Rv1932|Cfp20|Tpx	x	x	
Rv1980c|Mpt64	x	x	
Rv2031c|HspX	x	x	x
Rv2220|GlnA1	x	x	
Rv2244|AcpM	x	x	x
Rv2376c|Cfp2	x	x	
Rv2626c			x
Rv2780|Ald	x	x	
Rv2878c|Mpt53	x	x	
Rv3248c|SahH	x	x	
Rv3418c|GroES	x	x	
Rv3441c|MrsA			x
Rv3803c|Mpt51|fbpD	x	x	
Rv3804c|Ag85a	x	x	
Rv3841|BfrB	x	x	
Rv3874|CFP10			
Rv3875|EsxA|Esat6	x		

1 [12] ^2^ [13], [39].

Subsequent experiments evaluating the content of exosomes isolated from the BAL fluid from *Mtb*-infected BALB/c mice, revealed numerous additional potential protein candidates and corroborated the 25 proteins originally identified from our macrophage results, as well as generated dozens of additional candidates, 5 of which were included in this study (Table 1) ([13] and unpublished data). This trial confirmed that BAL fluid, derived from the site of the infection, was a rich source of mycobacterial proteins. However, despite the value of BAL fluid in the biomarker discovery pipeline, due to the invasive nature of BAL fluid collection (on par with sputum induction), this fluid is not ideal for a point of care platform.

Lastly, sera from a representative sample (“discovery group”) of eight Ugandans diagnosed with active pulmonary tuberculosis were collected. From this sera, exosomes were harvested and mined for additional candidate biomarkers by LC tandem MS. Compared to the macrophage and mouse exosome samples, the human samples were significantly more complex, an attribute of the abundance of host protein in sera-exosomes. While only 2 additional proteins were included from this endeavor, the potential relevance of these proteins drives their inclusion in our MRM assays. Of equal importance, this effort also served to confirm several of the proteins previously identified (Table 1) and included in the development of our MRM assays.

### Adaptation of Shotgun Proteomics to Targeted Analysis

From the comprehensive list of potential biomarker candidates from our discovery experiments, two hundred and nine peptides from thirty-six proteins underwent SRM development (Figure 1). Initially, the LC-MS/MS spectra from the discovery experiments were interrogated for the most intense transition ions. When data was not available for multiple peptides from each protein, *in* *silico* analysis was performed to predict which peptides and transition ions would be most suitable for successful SRM development. One hundred and six peptides remained following initial SRM development and were multiplexed into twenty-four 45-minute methods (Figure 1), which were further evaluated utilizing exosomes isolated from biofluids collected from the guinea pig model system of TB infection as a relevant matrix (see Methods). All MRM methods were screened and several peptides were removed due to peak interference with exosome background (Figure 1). In addition, all peptides were screened for potential interfering peptides using the SRM Collider software (Table S3). Two peptides showed potential interference as a result of this analysis. Lastly, to increase the confidence of results, when possible multiple peptides were chosen for each protein. Ultimately, 76 peptides from 33 proteins remained for multiplexing (Table 1) into a final assay set that included three 15-minute and fourteen 24-minute, scheduled MRM assays, allowing us to run approximately 1 patient sample per day.

### Interrogation of Clinical Samples

The finalized multiplex MRM assays were applied to a blinded, randomized sample set (n = 59) of exosomes isolated from serum from the “qualification group”. Subjects included those diagnosed with either smear-positive or smear-negative TB (TB+) (n = 41), or as not having active TB (TB−) (n = 18) (Figure S1). The TB+ patients were further subcategorized as having either pulmonary (n = 31) or extrapulmonary disease (n = 10). The TB- control patients could be categorized as either having no evidence of exposure to *Mtb* (n = 9) or as having latent TB infection (LTBI) (n = 9). In general, patients were young, with a high prevalence of advanced, untreated HIV (Table S1).

### Interpretation of MRM analyses

**Figure 2:**
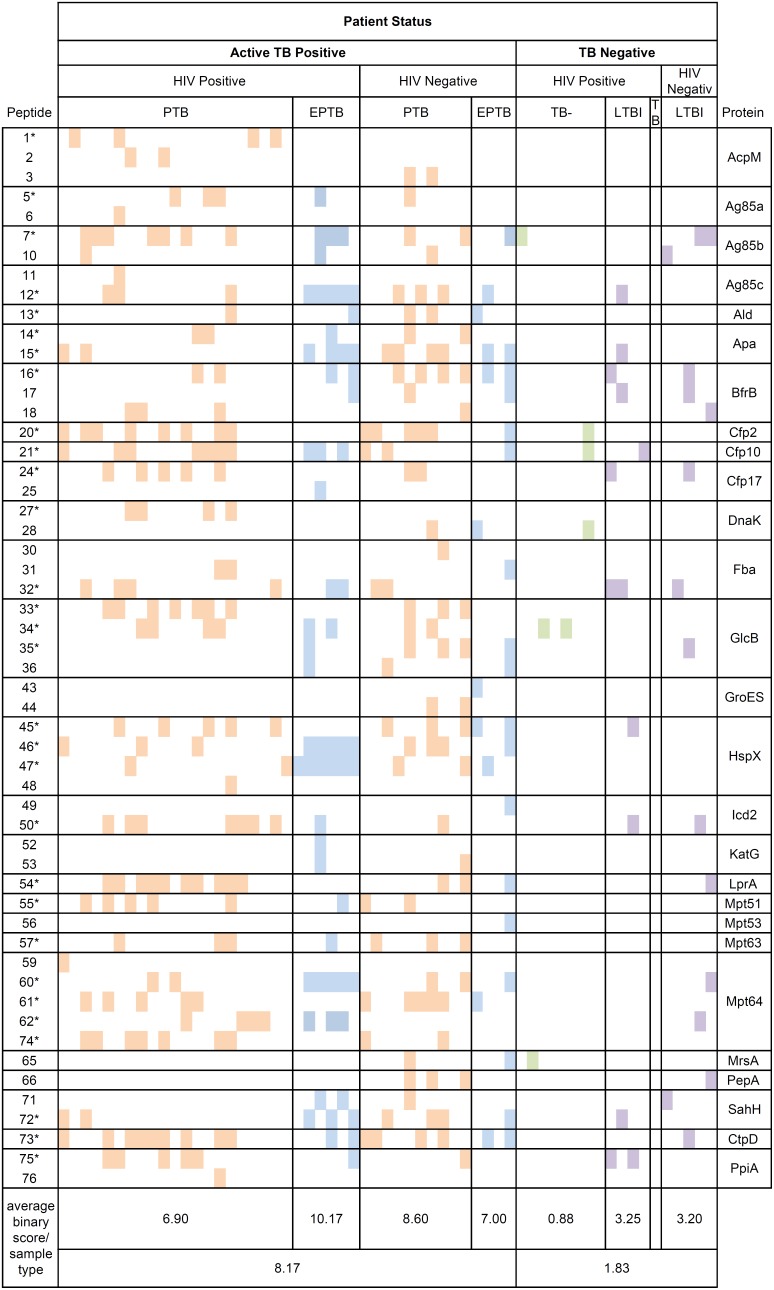
Summary of Peptides Detected by MRM. Colored boxes signify the presence of a peptide above the positive cut-off in samples from EPTB (blue), LTBI (purple), TB− (green) and PTB (orange) categories. Presence of peptides was determined using a binary metric and those peptides which failed to yield a signal above background are left blank. Peptides moving ahead to validation assay are indicated by *.

The presence of each of the seventy-six peptides was evaluated in each patient sample. Binary scores were assigned (peptide present = 1, peptide absent = 0) to compare raw peak values against the unique normal reference limit determined for each peptide; these results are summarized in Figure 2, in which a positive peptide identification is represented by a color box. Based on the analysis, Twenty-two peptides were not detected above the normal reference level in any patient sample, and thus were not considered in our follow up analyses or included in Figure 2.

**Figure 3:**
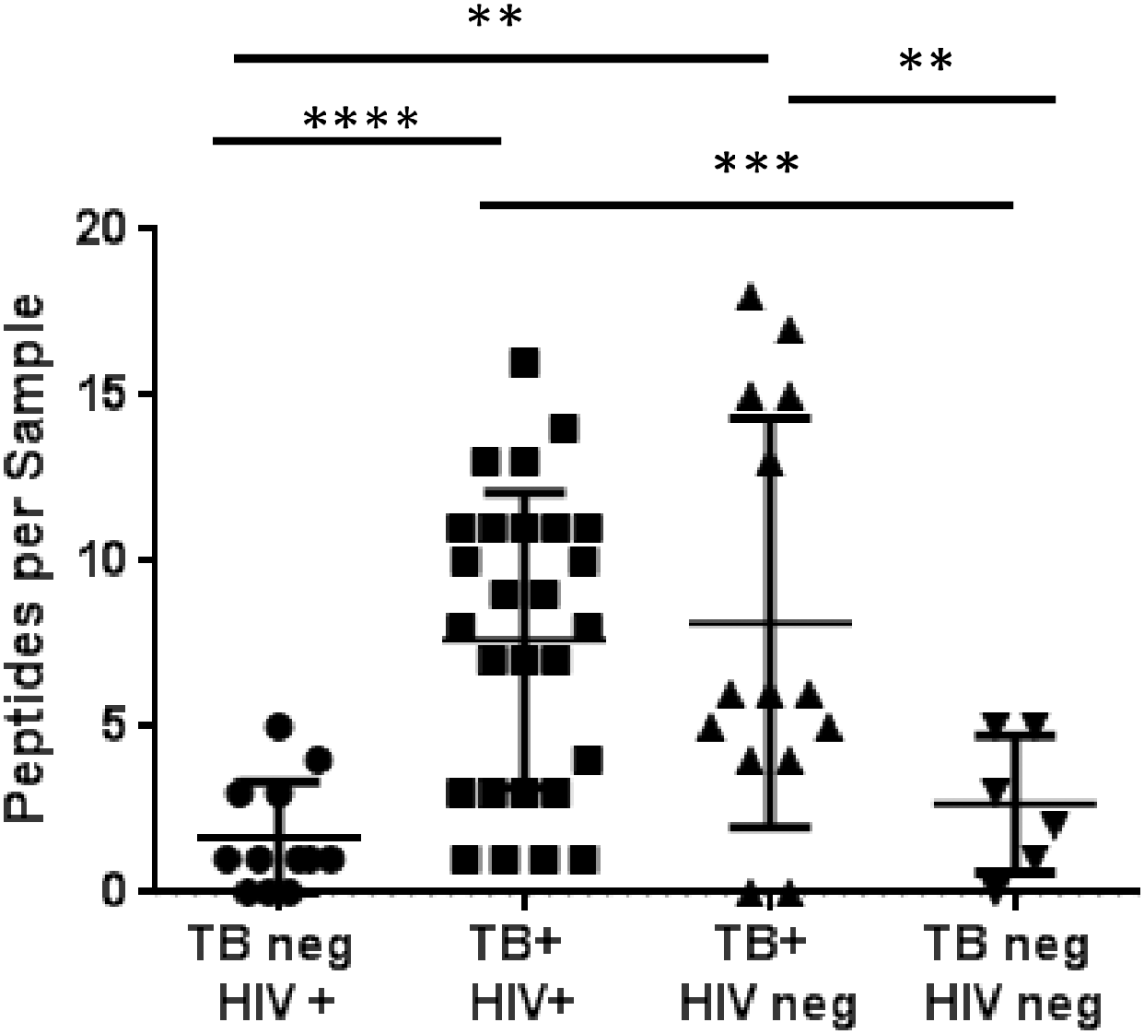
Scatter plot showing the total number of peptides identified in each sample class broken down by TB and HIV status. P-values from unpaired t-tests are represented as follows: **p<0.01, ***p<0.001, ****p<0.0001.

The proportion of TB+ patients exceeding the normal reference limit ranged between 0–42% for a given peptide. All but two patients with active TB (PTB and EPTB) contained at least one peptide marker (95%), ranging from 0 to 17 peptides identified (Figure 3). The average number of peptides detected per patient was consistent (∼8) irrespective of the site of the infection, however, the peptides represented in PTB and EPTB samples did vary, including those found as significantly different between TB and non-TB patients (Table 2). The proportion of TB patients with a positive MRM assay did not vary significantly by smear status; no significant differences were seen between the number or identity of the peptides found in smear-negative (average of 7.43 peptides) TB samples when compared to the smear-positive (7.46 peptides) TB samples. Similarly, HIV status did not affect the number of peptides observed among TB samples, with an average of 7.63 and 8.14 peptides observed in HIV+ versus HIV- patients (Figure 3). Twenty-nine peptides from 17 proteins were unique to those with active TB, including: AcpM, Ag85a, Ald, DnaK, GroES, Mpt51, Mpt53, Mpt63 and MrsA (Figure 2). In the cohort of 41 TB+ patients screened, 83% could be positively diagnosed using a biomarker candidate panel of 7 peptides (Table 3A); with at least one of these peptides present in 81% of PTB and 90% of the EPTB patients. The addition of 2 extra peptides (Table 3B) to the diagnostic panel increases the identification rate to 90% of all patients with either pulmonary or extrapulmonary active TB. While no single peptide or protein uniquely identified LTBI patients from TB patients or suspects (Figure 2), several proteins, including Ag85b, BfrB, Fba, and Mpt64 were found in TB+ and LTBI+ patient samples (Figure 2). Interestingly, of the 4 TB+ patients that could not be identified with the panel of 9 candidate peptide biomarkers, 2 could be identified by the addition of one more Mpt64 peptide, which was also found in LTBI serum samples, while 2 TB+ samples could not be identified by any of the 76 original candidate peptides.

**Table 2:** Peptides that distinguish (A) PTB or (B) EPTB patients from non-TB patients.

A) Peptide	Sequence	Protein	*p*-value^1^
33	FALNAANAR	GlcB	0.0361
61	VYQNAGGTHPTTTYK	Mpt64	0.0505
62	AFDWDQAYR	Mpt64	0.0448
74	EAPYELNITSATYQSAIPPR	Mpt64	0.0428
**B) Peptide**	**Sequence**	**Protein**	***p*** **-value** 2
7	PGLPVEYLQVPSPSMGR	Ag85	0.0367
12	FLEGLTLR	Ag85c	0.0169
15	LYASAEATDSK	Mpt32	0.0054
36	ATIEQLLTIPLAK	GlcB	0.0297
46	DGQLTIK	HspX	0.0006
47	SEFAYGSFVR	HspX	0.0007

1 Chi-squared test comparing raw peak areas of PTB patients to non-TB patients.

2 Chi-squared test comparing raw peak areas of EPTB patients to non-TB patients.

**Table 3:** Peptides specifically detected in active TB patients.

A) Peptide	Sequence	Protein	Present in % of TB+ samples
33	FALNAANAR	GlcB	24
46	DGQLTIK	HspX	29
47	SEFAYGSFVR	HspX	27
55	WHDPWVHASLLAQNNTR	Mpt51	20
57	GSVTPAVSQFNAR	Mpt63	17
61	VYQNAGGTHPTTTYK	Mpt64	27
74	EAPYELNITSATYQSAIPPR	Mpt64	22
**B) Peptide**	**Sequence**	**Protein**	**Present in % of TB+ samples**
1	IPDEDLAGLR	AcpM	10
36	ATIEQLLTIPLAK	GlcB	7

(A) Peptides found in 80% of TB+ samples examined. (B) Peptides found in 90% of the TB+ samples examined.

### Exquisite Detection of *Mtb* Proteins

Our assays used multiple peptides for each protein whenever possible in order to increase the likelihood that the protein detected was a bona fide *Mtb* protein. Nonetheless, 10 of our 33 candidate biomarker proteins were monitored by a single peptide due to the inability to generate a successful SRM assay for other tryptic peptides within these proteins. The remaining 23 proteins were monitored by 2–5 peptides. Nineteen of the 41 TB+ patients (46%) contained at least one protein identified by multiple peptides. Of the 19 patients, 9 (47.4%), 5 (26.3%) and 5 (26.3%), had one, two and three proteins identified by multiple peptides, for a total of 34 occurrences. Proteins with this distinction include: Ag85b, Ag85c, Apa, BfrB, GlcB, HspX, KatG, and Mpt64; this concordance resulted in increased confidence in the utility of these proteins as TB biomarkers (Table 4).

**Table 4:** Mycobacterial proteins identified by multiple peptides in TB patient samples.

Proteins	RepresentativePeptide (#)	Samples with ≥ 1peptide (n)	Samples with ≥ 1peptide (%)	Samples with ≥ 2peptides (n)	Samples with ≥ 2peptides (%)
AcpM	1, 2, 3	8	19.5	0	0.0
Ag85a	5, 6	6	14.6	0	0.0
Ag85b	7, 10	14	34.1	2	**14.3**
Ag85c	11, 12	12	29.3	1	**8.3**
Apa	14, 15	16	39.0	1	**6.3**
BfrB	16, 17, 18	13	31.7	4	**30.8**
Cfp17	24, 25	8	19.5	0	0.0
DnaK	27, 28	6	14.6	0	0.0
Fba	30, 31, 32	12	29.3	0	0.0
GlcB	33, 34, 35, 36	17	41.5	7	**41.2**
GroES	43, 44	3	7.3	0	0.0
HspX	45, 46, 47, 48	25	61.0	9	**36.0**
Icd2	49, 50	10	24.4	0	0.0
KatG	52, 53	2	4.9	1	**50.0**
Mpt64	59, 60, 61, 62, 74	29	70.7	9	**31.0**
SahH	71, 72	12	29.3	0	0.0
PpiA	75, 76	8	19.5	0	0.0

## Discussion

Identifying a biomarker panel for diagnosis of active TB is of paramount importance. Towards this goal, we generated multiplex peptide bioassays for 76 peptides representing 33 proteins initially discovered in exosomes by shotgun proteomics. The aim of this study was to identify biomarker candidates and optimize MRM methods so that screening and validation could be expanded to larger sample populations. The work described here provides the foundation for qualifying exosome-based *Mtb*-specific biomarkers for the detection of active TB using an MRM-MS platform. MRM-MS is a technique with high analytic sensitivity and specificity which takes advantage of a triple quadrupole MS system. Designing MRM assays requires *a priori* knowledge of proteins and peptides of interest, from which a combination of unique precursor/transition ion pairs allows us to confidently identify the target at low femtomolar/high attomolar levels in a complex matrix [26]. In addition to its exquisite sensitivity, MRM-MS has several additional advantages over traditional proteomic screening assays, namely, the ability to identify dozens of analytes in a single assay, allowing for more data to be collected from samples of limited quantity, as well as the ability to confirm or validate discovery efforts without the reliance on available immune reagents. Further, MRM assays can be applied to samples that were eliminated from shotgun or discovery platforms due to interference of contaminating proteins. This aspect is exemplified by our human discovery efforts, in which only 8 *Mtb* proteins (including 2 novel proteins) were confidently identified by LC-MS/MS and included in our multiplex MRM analyses. In our MRM assays, proteins originating from the host are disregarded and only peptides of interest belonging to *Mtb* are selected for fragmentation and detection; this enables us to mine for and generate targeted peptide bioassays, which, with the addition of isotopic peptide standards, can be used to validate biomarker candidates identified in multiple original discovery experiments [27]–[30]. From our analyses, 20 of the 33 proteins identified during the discovery phase were confidently identified in human sera and will proceed to the validation phase.

Sixteen of these proteins were identified in our preliminary sample set of LTBI patient exosomes. While it is unclear how robust this initial finding is given the small sample size and discovery aspects of this work, it is tantalizing to hypothesize that the identification of these proteins represents key physiological changes in dormant bacilli–reflecting adaptation to the dynamic host environment or, perhaps, the early stages of reactivation. We have shown that this MRM assay may indeed be more sensitive than smear microscopy; whether it is more sensitive than culture has yet to be fully established. It may be necessary to reevaluate the LTBI designation if the number of TB peptides exceeds a certain threshold; these individuals may have a bacterial infection, however not have bacilli replicating at sufficient levels for detection of AFB by smear or culture. Early treatment in these individuals may be beneficial to preventing progression of disease, as well as controlling the spread of infection to others in close contact with the initial suspect. Further studies surveying larger cohorts of LTBI patients are underway to address these intriguing possibilities.

MRM MS assays have limitations including the contribution of confounding results due to detection of similar host peptides in assays. The utilization of serum exosomes eliminates most host serum components, reduces the concentration of confounding serum proteins, and thereby increases the overall ratio of bacterial to host components. However, over 99% of the remaining proteins are still host derived. Therefore, the host protein content in exosomes will differ amongst samples, and may result in false positive data signals that cannot be excluded or subtracted despite optimization of our assays in sample matrixes from animal models and healthy donors. Similarly, while detecting a peptide indicates its presence, not detecting a peptide does not indicate its absence. With this in mind, we included in this study a diverse sample population, in order to determine the effect disease and health status has on the detection of selected individual Mtb peptides/proteins. The data presented here suggests that detection of *Mtb* biomarkers in exosomes does not rely on the immunocompetence of the host, as no difference was observed amongst the Mtb peptides detected between patients co-infected with HIV (*p* = 0.7073). From this we hypothesize that there may be a potential added benefit to exosome-based applications over current diagnostics that are adversely impacted by immunosuppressed states. We anticipate that a panel of proteins capable of identifying TB irrespective of infection site, additional health factors or age will emerge resultant of additional studies and validation assays in larger sample cohorts.

Analysis of our results led to the reduction of candidate serum biomarkers from 76 to 30 peptides. Based on the triage of candidate biomarker peptides in this study, expansion of MRM studies to larger sample sets is now much more feasible. These final 30 candidates will be combined into an MRM assay that will enable relatively high throughput screening of exosomes from TB patients, reducing our per patient sample processing and run time from ∼26 hours to 1–2 hours, therefore allowing us to expand our investigations on these promising biomarkers. In addition, these assays may be used to address questions regarding pathogenesis of *Mtb*, possibly explaining the diverse disease spectrum observed in TB patients. Preliminary analysis of a small sample of patients suggests the ability to stratify disease stage by biomarker detection, particularly between EPTB and TB patients. Clearly there was variation in the type of proteins identified in the MRM between patient populations especially when comparing pulmonary TB to EPTB. Differences in host genetics, strains of mycobacteria, time post-infection, state of the immune response, etc. will all lead to changes in mycobacterial protein expression and therefore changes in mycobacterial proteins present in exosomes. Nevertheless, expansion of these studies may provide additional support for specific biomarkers to understand variations in disease manifestations, and may be further exploited to identify LTBI patients at risk for developing active disease. Further, this report deployed samples derived from a clinic in Uganda for our preliminary evaluation. While this is an appropriate starting-point for assay development, samples from other geographical regions should be included in future studies to address the effects of antigenic variation between *Mtb* clades and its influence on the exo-proteome profile [26], [31].

In this study, MRM-MS assays were developed and applied to a simplified fraction of serum to detect only mycobacterial proteins enriched within exosomes. Twenty of the 33 proteins targeted for detection were found in TB patient exosomes, including the detection of multiple peptides from 8 well-recognized *Mtb* proteins (Antigen 85B, Antigen 85C, Apa, BfrB, GlcB, HspX, KatG, and Mpt64). Four of these proteins, Antigen 85B, Antigen 85C, Apa, and GlcB, are known adhesins [32] and may therefore be targeted to host exosomes based on their physiologic interactions with host cells upon intracellular invasion by *Mtb*. In addition, the remaining four proteins, BfrB, HspX, KatG, and Mpt64, are associated with mycobacterial virulence [33], with BrbB and KatG specifically associated with oxidative stress and nitrogen responses [34], and these two proteins along with Mpt64 are implicated as essential for intracellular survival of Mtb and potentially its persistence in host cells [35]–[38]. Thus, their association with host exosomes may be associated with the function of these proteins during establishment and maintenance of an intracellular infection; future studies are planned to address these questions. From a practical biomarker standpoint, the proteins/peptides identified in this study will be used in second generation MRM MS assays to assess a larger cohort of well-characterized patients in order to develop a blood-based “biosignature” that can be used as a diagnostic for active TB.

## Supporting Information

Figure S1 **Flow diagram describing patient enrollment at Mulago Hospital, Kampala, Uganda.**
(PDF)

Table S1 **Demographic and baseline clinical characteristics of Ugandan patients in the TB evaluation** **sample.**
(PDF)

Table S2 **Complete list of Proteins/Peptides included in the seventeen final MRM assays, including all transitions monitored and assay parameters.**
(PDF)

Table S3 **SRM Collider Output. All Peptides were queried against a library of human tryptic peptides to determine potential sources of matrix interference.**
(PDF)

Document S1 **Scaffold Output for shotgun LC-MS/MS discovery in eight patients with active pulmonary tuberculosis.**
(XLSX)

Methods S1 **Patients were sampled from the overall cohort of patients with unexplained cough for ≥2 weeks, based on the reference standard definitions for TB outcome classification described in the supplemental methods section.**
(PDF)
