# Supplementary figures and images for: Detection of Mycobacterium tuberculosis Peptides in the Exosomes of Patients with Active and Latent M. tuberculosis Infection Using MRM-MS
Source: PLoS One. 2014 Jul 31;9(7):e103811. doi: 10.1371/journal.pone.0103811 (PMC4117584; doi:10.1371/journal.pone.0103811)

**Supplementary Figure 1:**

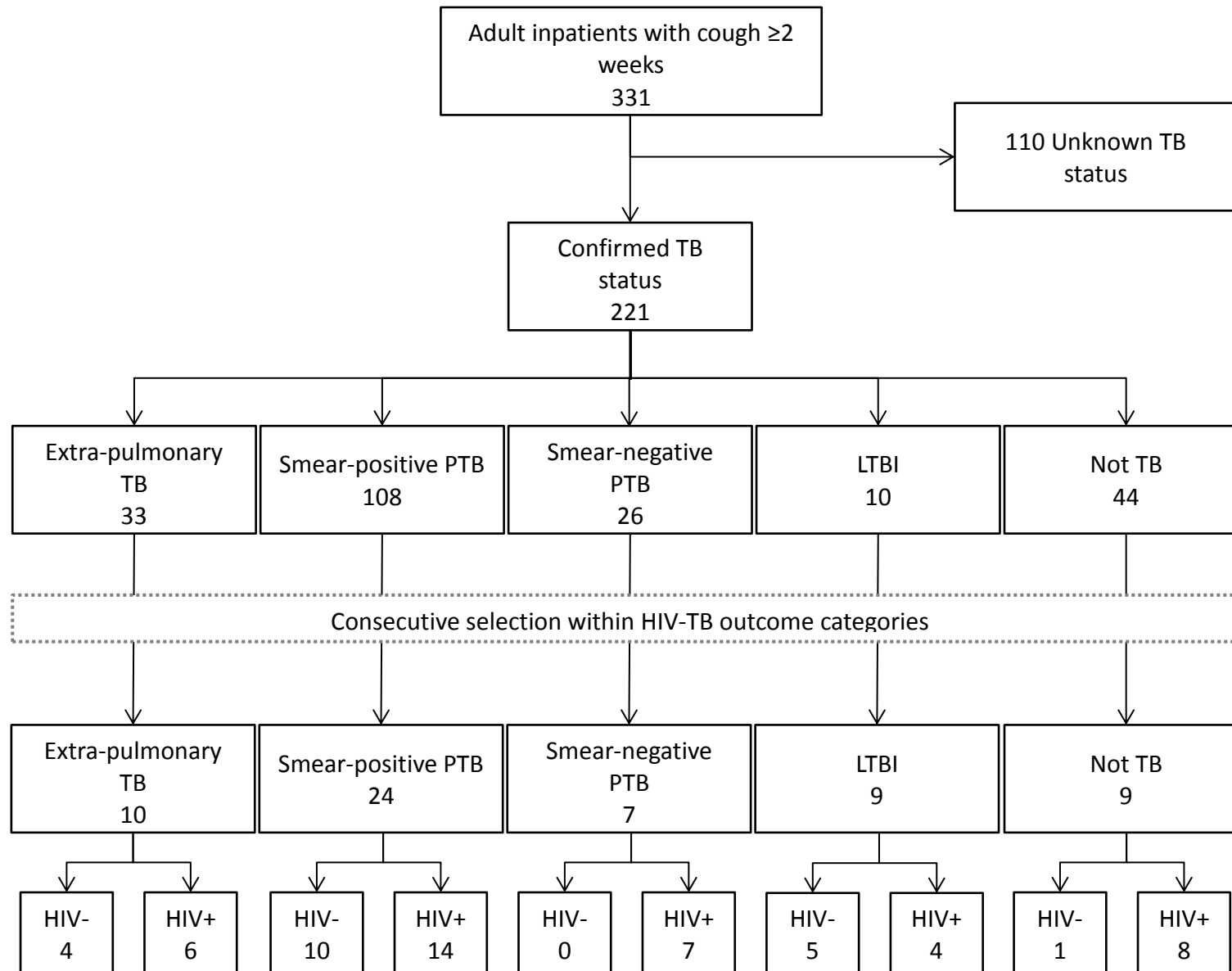

Supplement: Figure S1 — Flow diagram describing patient enrollment at Mulago Hospital, Kampala, Uganda. (PDF) [file pone.0103811.s001.pdf]
