# Supplementary material for: Detection of Mycobacterium tuberculosis Peptides in the Exosomes of Patients with Active and Latent M. tuberculosis Infection Using MRM-MS
Source: PLoS One. 2014 Jul 31;9(7):e103811. doi: 10.1371/journal.pone.0103811 (PMC4117584; doi:10.1371/journal.pone.0103811)
Supplement: Table S1 — Demographic and baseline clinical characteristics of Ugandan patients in the TB evaluation sample. (PDF) [file pone.0103811.s002.pdf]

Supplementary Table 1:

**Characteristics**<br>**N (%)**	**EPTB**<br>(n=10)	**PTB**<br>(n=31)	**LTBI**<br>(n=9)	**Not TB**<br>(n=9)
Female sex	4 (40)	19 (61)	4 (44)	3 (33)
Median age (IQR), years	34 (26-41)	29 (23-35)	31 (25-40)	35 (21-35)
HIV-positive	6 (60)	21 (68)	4 (44)	8 (89)
Median CD4+ count (IQR), cells/µL	18 (9-39)	252 (113-337)	32 (17-392)	190 (71-240)
Antiretroviral therapy*	3 (14)	0 (0)	3 (75)	0 (0)
CTX prophylaxis†	19 (90)	6 (100)	4 (100)	7 (88)
Antibiotics for current illness	5 (50)	24 (77)	4 (44)	6 (67)
Dyspnea	8 (80)	19 (61)	5 (56)	5 (56)
Fever, chills, night sweats	10 (100)	31 (100)	9 (100)	9 (100)
Weight loss (>5 kg) ‡	10 (100)	20 (65)	6 (67)	3 (33)
Cough in past 7 days	8 (80)	30 (97)	7 (78)	8 (89)
Smear-positive	1 (10)	24 (77)	0 (0)	0 (0)
Mycobacterial culture-positive	3 (30)	31 (100)	0 (0)	0 (0)

Abbreviations: IQR, Interquartile range; N, number; PTB, Pulmonary tuberculosis; EPTB, Extra-pulmonary tuberculosis; LTBI, Latent tuberculosis infection; TB, Tuberculosis; HIV, Human immunodeficiency virus; CTX, Co-trimoxazole prophylaxis; kg, kilograms
Legend: * 14 missing values (9 PTB, 1 LTBI, 4 Not TB), † 1 missing value (PTB), ‡ 7 missing values (5 PTB, 1 LTBI, 1 Not TB)
