# Supplementary material for: Detection of Mycobacterium tuberculosis Peptides in the Exosomes of Patients with Active and Latent M. tuberculosis Infection Using MRM-MS
Source: PLoS One. 2014 Jul 31;9(7):e103811. doi: 10.1371/journal.pone.0103811 (PMC4117584; doi:10.1371/journal.pone.0103811)
Supplement: Table S2 — Complete list of Proteins/Peptides included in the seventeen final MRM assays, including all transitions monitored and assay parameters. (PDF) [file pone.0103811.s003.pdf]

Gene /Protein Name	Peptide Sequence (z)	Retention Time Start (min)	Retention Time End (min)	[M+H]	Average Mass [m/z]	Transition	Dwell Time (sec)	Cone Voltage (V)	Collision Energy (V)
**Rv0009/PpiA**	IALFGNHAPK(3)	6.56	7.56	1067.61	356.54	452.26	0.003	35	18
						566.30	0.003	35	12
						623.33	0.003	35	17
	VIQGFMIQGGDPTGTGR(2)	7.5	8.5	1732.87	867.44	588.31	0.006	35	26
						945.44	0.006	35	30
						1058.52	0.006	35	26
**Rv0066c/Icd2**	GISNFHSPSDVIVDASMPAMIR(3)	7.74	8.74	2344.16	782.05	587.33	0.008	35	29
						876.44	0.008	35	34
						991.47	0.008	35	35
	LPNISASVPQLVAAIK(2)	11.44	13.44	1619.98	810.99	839.53	0.019	35	27
						938.60	0.019	35	30
						1025.64	0.019	35	22
**Rv0125/PepA**	TQDVAVLQLR(2)	7.11	8.11	1141.66	571.83	628.41	0.007	35	17
						699.45	0.007	35	15
						798.52	0.007	35	19
	TQDVAVLQLR(2)	5	6	1141.66	571.83	628.41	0.007	35	17
						699.45	0.007	35	15
						798.52	0.007	35	19
**Rv0129c/Ag85c**	FLEGLTLR(2)	8.4	9.04	949.56	474.78	502.33	0.008	35	12
						559.36	0.008	35	18
						688.40	0.008	35	16
	VQFQGGGPHAVYLLDGLR(3)	8.5	10.5	1927.03	643.01	686.42	0.008	35	25
						849.48	0.008	35	24
						948.55	0.008	35	24
**Rv0350/DnaK**	TTPSIVAFAR(2)	7.7	8.7	1061.60	531.80	563.33	0.007	35	17
						763.45	0.007	35	24
						860.50	0.007	35	17
	ITQDLLDR(2)	6.03	7.03	972.54	487.27	516.31	0.003	35	13
						631.34	0.003	35	18
						759.40	0.003	35	16
**Rv0363c/Fba**	LYTSPEDFEK(2)	5.58	6.58	1227.58	614.79	667.29	0.003	35	16
						764.35	0.003	35	18
						851.38	0.003	35	22
	SEIEEALR(2)	5.99	6.89	945.49	473.75	488.28	0.003	35	22
						617.33	0.003	35	16
						730.41	0.003	35	13
	LRPDILAQGQQVAAAK(3)	5.7	6.7	1678.97	560.32	587.35	0.003	35	22
						715.41	0.003	35	22
						772.43	0.003	35	29
**Rv0440/GroEL2**	AAVEEGIVAGGGVTLLQAAPTLDELK(2)	7.74	8.54	2523.78	1261.69	815.45	0.006	35	46
						1311.75	0.006	35	40
						1412.80	0.006	35	40
**Rv0931c/PstS1**	ASFLDQVHFQPLPPAVVK(3)	10.5	12.5	1995.15	665.04	513.34	0.03	35	35
						610.39	0.03	35	34
						820.53	0.03	35	40
	GLGEAQLGNSSGNFLLPDAQSIQAAAAG FASK(3)	13	15	3090.56	1030.85	1034.56	0.019	35	38
						1121.59	0.019	35	37
						1249.65	0.019	35	44
	SDGSGDTFLFTQYLSK(2)	10	11	1764.84	883.42	886.47	0.03	35	28
						999.55	0.03	35	34
						1146.62	0.03	35	24
**Rv1270c/LprA**	ITGNSSADDIATLAGSR(2)	6.47	7.47	1647.82	824.91	903.49	0.003	35	23
						1018.52	0.003	35	33
						1089.55	0.003	35	32
**Rv1469/CtpD**	VVAASELVVGDR(2)	6.66	7.66	1213.68	607.84	658.39	0.006	35	21
						787.43	0.006	35	17
						874.46	0.006	35	16
**Rv1827/GarA**	LVFLTGPK(2)	7.52	8.52	873.55	437.77	515.32	0.007	35	13
						662.39	0.007	35	16
						761.46	0.007	35	11
	HPDSDIFLDDVTVSR(3)	7.44	8.44	1715.84	572.61	676.36	0.006	35	21
						791.39	0.006	35	22
						904.47	0.006	35	27
**Rv1837c/GlcB**	ATIEQLLTIPLAK(2)	13.5	15.5	1411.86	705.93	428.29	0.063	35	21
						755.50	0.063	35	21
						868.59	0.063	35	29
	NYTAPGGGQFTLPGR(2)	5.89	6.89	1535.67	768.84	329.19	0.003	35	24
						932.49	0.003	35	26
						1086.57	0.003	35	26

Gene /Protein Name	Peptide Sequence (z)	Retention Time Start (min)	Retention Time End (min)	[M+H]	Average Mass [m/z]	Transition	Dwell Time (sec)	Cone Voltage (V)	Collision Energy (V)
**Rv1837c/GlcB**	VVFINTGFLDR(2)	9.5	11.5	1279.71	640.85	708.37	0.019	35	19
						822.41	0.019	35	18
						935.49	0.019	35	23
	FALNAANAR(2)	5.91	6.91	946.51	474.26	502.27	0.003	35	21
						616.32	0.003	35	17
						729.40	0.003	35	12
**Rv1860/Apa**	TTGDPPFPGQPPPVANDTR(2)	6.57	7.57	1962.96	982.48	869.45	0.003	35	32
						966.50	0.003	35	32
						1248.63	0.003	35	36
**Rv1886c/Ag85b**	PGLPVEYLQVPSPSMGR(3)	9	11	1826.96	609.65	547.27	0.03	35	25
						731.35	0.03	35	21
						830.42	0.03	35	29
	WETFLTSELPQWLSANR(2)	15.95	16.95	2077.04	1039.52	971.51	0.063	35	34
						1084.59	0.063	35	34
						1300.66	0.063	35	34
	NDPTQQIPK(2)	5.4	6.5	1039.54	520.77	613.37	0.005	35	18
						714.41	0.005	35	18
						811.47	0.005	35	18
**Rv1908c/KatG**	EATWLGDER(2)	6.41	7.41	1076.51	538.75	589.29	0.003	35	16
						775.37	0.003	35	19
						876.42	0.003	35	15
	FAPLNSWPDNASLDK(2)	8.06	9.06	1674.82	837.91	859.42	0.008	35	25
						1045.49	0.008	35	28
						1132.53	0.008	35	26
	TFGFGFGR(2)	8.09	10.09	887.44	444.72	583.30	0.011	35	17
						640.32	0.011	35	15
						787.39	0.011	35	15
**Rv1926c/Mpt63**	GSVTPAVSQFNAR(2)	6	7	1332.93	667.47	722.36	0.003	35	23
						821.43	0.003	35	26
						989.52	0.003	35	23
	TADGINYR(2)	5.2	6.2	908.45	455.22	565.31	0.003	35	14
						622.33	0.003	35	18
						737.36	0.003	35	14
**Rv1932/Tpx**	DSFGEDYGVTIADGPMAGLLAR(2)	7.96	8.96	2254.07	1128.04	828.48	0.011	35	35
						1184.65	0.011	35	36
						1285.69	0.011	35	38
**Rv1980c/Mpt64**	GTQAVVLK(2)	5.5	6.5	815.06	408.53	458.33	0.003	35	20
						529.37	0.003	35	10
						657.43	0.003	35	12
	VYQNAGGTHPTTTYK(3)	5.05	5.85	1637.81	546.60	613.32	0.005	35	24
						710.37	0.005	35	23
						847.43	0.005	35	20
	FLSAATSSTPR(2)	5.65	6.55	1136.80	569.40	648.33	0.003	35	23
						719.37	0.003	35	20
						790.41	0.003	35	16
	AFDWDQAYR(2)	7.08	8.08	1170.52	586.26	652.30	0.006	35	21
						838.38	0.006	35	17
						953.41	0.006	35	16
	SLENYIAQTR(2)	6.56	7.56	1193.62	597.81	751.41	0.006	35	17
						865.45	0.006	35	18
						994.50	0.006	35	19
	EAPYELNITSATYQSAIPPR(2)	7.97	8.97	2220.12	1111.06	1190.62	0.011	35	33
						1291.66	0.011	35	41
						1404.75	0.011	35	41
**Rv2031c/HspX**	AELPGVDPDK(2)	5.95	7.5	1141.53	520.77	359.19	0.003	35	18
						630.31	0.003	35	14
						727.36	0.003	35	15
	DGQLTIK(2)	5.64	6.44	773.44	387.72	474.33	0.003	35	11
						602.39	0.003	35	11
						659.41	0.003	35	12
	TVSLPVGADEDDIK(2)	6.85	7.75	1457.74	729.87	734.32	0.006	35	15
						862.38	0.006	35	15
						1058.50	0.006	35	15
**Rv2220/GlnA1**	GGYFPVAPNDQYVDLR(2)	7.96	8.96	1811.88	905.94	908.45	0.006	35	35
						1119.54	0.006	35	29
						1386.70	0.006	35	31
	IPITGSNPK(2)	5.43	6.43	925.54	463.77	603.31	0.005	35	17
						716.39	0.005	35	17
						813.45	0.005	35	13

Gene /Protein Name	Peptide Sequence (z)	Retention Time Start (min)	Retention Time End (min)	[M+H]	Average Mass [m/z]	Transition	Dwell Time (sec)	Cone Voltage (V)	Collision Energy (V)
**Rv2220/GlnA1**	LVPGYEAPINLVYSQR(2)	9.02	9.92	1817.98	909.99	992.55	0.013	35	28
						1089.61	0.013	35	34
						1160.64	0.013	35	32
	SVFDDGLAFDGSSIR(2)	8.57	9.57	1584.76	793.38	852.42	0.011	35	31
						965.51	0.011	35	22
						1022.53	0.011	35	23
**Rv2244/AcpM**	IESENPDAVANVQAR(2)	5.61	6.41	1611.80	806.90	828.47	0.005	35	22
						943.50	0.005	35	31
						1040.55	0.005	35	29
	LEEENPEAAQALR(2)	5.83	6.63	1469.30	735.65	758.42	0.003	35	30
						855.47	0.003	35	30
						969.51	0.003	35	31
	IPDEDLAGLR(2)	6.75	7.55	1097.59	549.79	773.42	0.006	35	19
						888.44	0.006	35	19
						985.49	0.006	35	19
	TVGDVVAYIQK(2)	6.08	7.08	1191.66	596.83	622.36	0.003	35	20
						721.42	0.003	35	17
						820.49	0.003	35	18
**Rv2376c/Cfp2**	GSLVEGGIGGTEAR(2)	5.42	6.92	1301.67	651.84	703.37	0.003	35	19
						760.39	0.003	35	22
						817.42	0.003	35	21
	SLADPNVSFANK(2)	5.2	6.2	1261.64	631.82	665.36	0.003	35	20
						779.40	0.003	35	23
						876.46	0.003	35	19
**Rv2626c/Hrp1**	DSIYYVDANASIQEMLNVMEEHQVR(3)	7.1	8.1	2956.83	985.46	1027.50	0.006	35	45
						1141.54	0.006	35	42
						1254.63	0.006	35	35
	GLAAGLDPNTATAGELAR(3)	5.97	6.53	1697.90	566.63	616.34	0.003	35	22
						717.39	0.003	35	22
						788.43	0.003	35	29
**Rv2780/Ald**	GLSTHEGALLSER(3)	5.79	6.79	1370.22	457.41	504.28	0.003	35	17
						617.36	0.003	35	18
						688.40	0.003	35	16
**RV2878c/Mpt53**	LQFTATTLSGAPFDGASLQGK(2)	8.87	10.87	2109.09	1055.54	1019.52	0.013	35	33
						1147.57	0.013	35	38
						1234.61	0.013	35	33
**Rv3248c/SahH**	EYAEVQPLK(2)	5.85	6.85	1076.57	538.78	357.25	0.003	35	17
						584.38	0.003	35	18
						784.46	0.003	35	17
	GVTEETTTGVLR(2)	5.1	6.1	1261.67	631.83	646.39	0.003	35	19
						747.44	0.003	35	23
						876.48	0.003	35	26
**Rv3418c/GroES**	DVLAVVSK(2)	6.55	7.55	831.55	415.75	432.28	0.003	35	20
						503.32	0.003	35	10
						616.40	0.003	35	12
	ILVQANEAETTTASGLVIPDTAK(3)	7.55	8.55	2342.26	781.42	531.28	0.006	35	30
						913.54	0.006	35	37
						1000.57	0.006	35	36
	RIPLDVAEGDTVIYSK(3)	7.05	8.05	1775.97	592.66	609.36	0.007	35	22
						710.41	0.007	35	23
						825.44	0.007	35	28
**Rv3441c/MrsA**	LAATVADAVSTAR(2)	6.13	7.13	1244.87	623.44	719.37	0.003	35	25
						790.41	0.003	35	19
						889.47	0.003	35	19
	VIAINAEPNGR(2)	5.54	6.54	1152.64	577.32	443.24	0.003	35	19
						757.36	0.003	35	23
						870.44	0.003	35	25
	YVLEELR(2)	5.69	6.69	920.51	461.26	546.29	0.003	35	14
						659.37	0.003	35	20
						758.44	0.003	35	17
**Rv3803c/FbpD**	WHDPWVHASLLAQNNTR(3)	5.82	6.82	2045.02	682.34	703.35	0.003	35	24
						816.43	0.003	35	24
						929.52	0.003	35	29
**Rv3804c/Ag85a**	FLEGFVR(2)	7.54	8.54	867.80	434.40	478.28	0.006	35	15
						607.32	0.006	35	15
						720.40	0.006	35	15
	NDPLLNVGK(2)	6.42	7.42	968.54	485.27	530.33	0.003	35	18
						643.41	0.003	35	18
						740.47	0.003	35	18

Gene /Protein Name	Peptide Sequence (z)	Retention Time Start (min)	Retention Time End (min)	[M+H]	Average Mass [m/z]	Transition	Dwell Time (sec)	Cone Voltage (V)	Collision Energy (V)
**Rv3804c/Ag85a**	ALGATPNTGPAPQGA(2)	5.75	6.65	1321.68	661.84	372.19	0.003	35	23
						540.28	0.003	35	23
						909.44	0.003	35	23
	VQFQSGGANSPALYLLDGLR(3)	13.06	14.16	2106.11	702.70	849.48	0.063	35	27
						962.57	0.063	35	27
						1130.66	0.063	35	33
**Rv3841/BfrB**	AGANLFELENFVAR(2)	11.5	13.5	1551.83	775.90	848.46	0.03	35	30
						977.51	0.03	35	23
						1124.57	0.03	35	22
	TVTDQVGR(2)	5.1	5.9	874.47	438.23	459.27	0.004	35	17
						574.29	0.004	35	14
						675.34	0.004	35	14
	EALALALDQER(2)	7.3	9.3	1227.66	614.83	660.33	0.006	35	18
						731.37	0.006	35	16
						844.45	0.006	35	22
**Rv3874/Cfp10**	GAAGTAAQAAVVR(2)	6.84	7.84	1143.64	571.82	643.39	0.003	35	25
						714.43	0.003	35	25
						785.46	0.003	35	16
	QELDEISTNIR(2)	6.8	7.8	1316.72	659.36	703.41	0.006	35	28
						832.45	0.006	35	25
						947.48	0.006	35	23
	TQIDQVESTAGSLQGQWR(3)	5.96	6.96	2003.99	668.66	674.34	0.003	35	30
						787.42	0.003	35	27
						874.45	0.003	35	25
**Rv3875/ESAT-6**	WDATATELNNALQNLAR(2)	10.87	12.87	1899.96	950.98	1013.55	0.03	35	31
						1126.63	0.03	35	31
						1255.68	0.03	35	31
