# Supplementary material for: Detection of Mycobacterium tuberculosis Peptides in the Exosomes of Patients with Active and Latent M. tuberculosis Infection Using MRM-MS
Source: PLoS One. 2014 Jul 31;9(7):e103811. doi: 10.1371/journal.pone.0103811 (PMC4117584; doi:10.1371/journal.pone.0103811)
Supplement: Table S3 — SRM Collider Output. All Peptides were queried against a library of human tryptic peptides to determine potential sources of matrix interference. (PDF) [file pone.0103811.s004.pdf]

	Potential Interfering Peptides (human tryptic)			
Mtb Peptide Sequence	Q1	RT	Human Peptide Sequence	Transitions
LPNISASVPQLVAAIK	809.75	30.44	TGSLKPNPASPLPASPYGGPTPASY	y8
	810.44	32.81	HNELTGDNVGPLILK	y10
	810.07	32.87	VITTHTSGWDSSPGAGFQVPEVR	y9
	810.73	33.12	WAETVRPEEVSQEHIETAYR	y8
	810.94	34.71	IDGLNVADIGLHDLR	y9
	811.1	34.73	QHNSGPNSKPVVSFIAGLTAPPGR	y8
	810.41	38.69	DSTVAVVVYDITNVNSFQQTTK	y8
QELDEISTNIR	659.33	20.25	DIIGDTSGHFQK	y6
	657.99	22.02	VSQTETGSWSAETAPGVHK	y6
	657.99	23.39	TQHLSVETSYLQHESGR	y6
	659.37	23.67	DVGAQILLHSHK	y8
	659.32	23.88	HFNSMSGPATLR	y7
	658.86	24.13	LATSLGASEQALR	y6
	658.33	24.63	AFHQWIQETR	y7
	658.96	24.83	YC[160]LEHGIQPDGQMPSDK	y6
	658.96	25.62	VVIC[160]HLPC[160]MNGGQC[160]SSR	y7
	658.36	25.8	EPISVSSEQVLK	y6
	658.98	26.25	EDLPNLESSEETEQINK	y6
	658.84	26.37	YFFTSVSRPGR	y7
	658.3	26.83	SSLSSNDDGYENIDSLTR	y8
	659.33	28.32	MC[160]LEANIPLEK	y7
	658	28.34	SQLLSNHQQQLQADSFK	y6
	658.86	28.37	QLSTALEGAASLR	y6
VIQGFMIQGGDPTGTGR	866.74	25.16	SSPPELPDVMKPQDSGSSANEQAVQ	y10
	867.77	26.04	TQPSSGVDSAVGTLPATSPQSTSVQAK	y10
	865.93	26.45	SELEEQLTPVAEETR	y6
	867.43	27.18	QISETNVILSMDNNR	y6
	867.43	27.58	MFIQTQDTPNPNSLK	y6
	866.42	27.8	VVVLMGSTSDLGHC[160]EK	y10
	865.94	29	VGPQYQAVVPDFDPAK	y11
	867.42	29.07	LSESGAIMTDLEENPK	y10
	865.93	29.23	VSDTVVEPYNATLSVH	y6
	867.42	29.93	WETPYMHALAAAASSK	y10
	866.93	29.98	LMAVQLLDSSNQEER	y11
	866.4	30.09	ESNEEEQVWHFLGK	y10
	865.94	31.03	VLNTGSDVEEAVADALK	y10
	867.47	32.58	YGLIYHASLVGQTSPK	y6
	866.75	32.96	VEKPTADAEAYVFTPNMIC[160]AGGEK	y6
	866.47	33.02	SGQGQGQFLIQQVTLK	y6
	867.47	33.53	ITFTPSSGIASEVTVPK	y6
	867.43	34.2	EEVVTVETWQEGSLK	y6
WHDPWVHASLLAQNNTR	682	35.38	NNQFQALLQYADPVSAQH	y8 y7 y6
	682.01	35.43	SGGGGDIHQGFQSLLTEVNK	y7 y6
	680.85	33.5	IC[160]EVINEAVWK	y8
	680.88	33.6	GLETAGGVMTALIK	y7
	682.38	33.69	YIAEAVASIVEAK	y7
	681.97	34.3	AWVWNTHADFADEC[160]PKP	y7
	682.03	34.51	IGQLQGEIIPTSFYHQGR	y7
	681.36	34.74	TGEIVLTQSPGTLSLSPGER	y8
	681.98	34.93	VSENDFEDLLSNQGFSSR	y8
	682.66	35.46	VEDAYILTC[160]NVSLEYEK	y7
	682.34	36.35	VSALLGEEDEEALHYLTR	y8
	681.66	36.75	ALDFEQEMATVASSSSLEK	y8
	682.31	37.79	GEYPDYQQWMGLSDSIR	y8
	682.35	37.83	SVDVFGSTPFQPFLTSTSK	y8
	682.69	38.17	ESLSQPGDFVLSVLSDQPK	y6
	682.37	38.22	ISGVGIDQPPYGIFVINQK	y7
	681.67	39.31	GFDGIPDNVDAALALPAHSY	y7
	682.36	39.52	ADHDVGSELPPEGVLGALLR	y8
	680.88	39.68	MNSVVALDLAISK	y8
	682.02	41.27	IGQIDGLISQLEMDQQAGK	y7
	681.38	41.3	SPSELFAQHIVTIVHHVK	y8
	681.01	42.32	HILYGC[160]SELFNATQFIK	y6
	682.67	43.12	ADAVQDSEMVELVELEIR	y7
	682.04	43.27	LHLIYLINDVLHHC[160]QR	y6
LRPDILAQGQQVAAAK	559.27	24.49	EEEDFHVDQVTTVK	y7 y6
	559.78	29.3	FYFENALSK	y8 y6
	559.26	29.61	GHPGDTPFGTFTLTGGN	y8 y7

	560.6	21.17	VLATLC[160]GQESTDTER	y7
	559.61	21.22	AIQLTYNPDESSKPN	y8
	559.27	21.24	VELEDWNGR	y8
	558.81	22.28	QTVSWAVTPK	y8
	559.92	24.32	FSPATHPSEGLEENY	y6
	560.58	24.48	SFTAVC[160]QDDGTWHR	y6
	558.82	24.72	SLGSAEALLQK	y8
	559.28	25.12	MILIQDGSQNTNVDK	y8
	560.63	25.41	DSLQASLRPHADELK	y7
	559.31	25.63	LQGSGVTVNALHPGVAR	y7
	558.76	26	SEQFTAMFR	y8
	560.24	26.05	EHFQSYDLDHMEK	y8
	559.91	26.81	AGPDLASC[160]LDVDEC[160]R	y8
	559.31	27.35	HINAQLNLDSLRPGK	y8
	559.29	27.56	EAALYPHLPPEADPR	y6
	559.29	28.52	EVESVDLPHC[160]HLIK	y8
	559.33	28.69	THHILIDLR	y7
	560.29	29.14	LLDYVATVEDEAAAAK	y6
	559.59	29.31	SISDNAYQYMLTDR	y6
	559.61	29.77	GMC[160]RPLALGGPGQVTY	y7
	559.94	30.37	GDVGMAGVAIDTVEDTK	y7
	559.27	30.53	MSSPC[160]HIEMILTEK	y8
WDATATELNNALQNLAR	950.13	43.24	ADTHDEILEGLNFNLTEIPEAQIHE	y10 y9
	949.51	36.9	AASDIWKPVLSIDTEPR	y10
	950.01	39.59	INESAEGEIIIDGINIAK	y11
	950.99	39.87	VLEGHTDFINGLVFDPK	y10
	950.86	40.92	PSLPSEPVVALDPIDPPGKPEVINITR	y10
	949.51	42.87	QEPSLGC[160]SIPAILFLPR	y10
	949.55	42.96	AGVASLLTTAEVVVTEIPK	y9
FAPLNSWPDNASLDK	836.75	31.8	TTPMDSTGVPHILEHTVLC[160]GSQK	y9 y8
	836.77	36.3	IPQSHIQQIC[160]ETILTSGENLAR	y10 y9
	837.94	26.56	NGVAQEPVHLDSPAIK	y9
	837.45	26.84	FVQEVVQSQQVAVGR	y9
	836.96	27.72	VELQAQTTTLEQAIK	y10
	837.39	31.48	QWVDTDDTSSENTVVPPETYVK	y10
	836.72	34.47	VTYHPDGPEGQAYDVDFTPPFR	y9
	837.97	34.7	HIGKPLLGGPFSLTTH	y8
	837.4	35.82	NIC[160]WGTQSKPLYETIEDNDVK	y10
TTGDPPFPGQPPPVANDTR	982.42	25.7	C[160]EEVIPDEEFDPQNSR	y12
	981.51	26.58	HSSHGSDVSLSQILKPNR	y8
SLADPNVSFANK	630.89	19.49	DYHFEC[160]YHC[160]EDC[160]R	y8
	631.3	20.45	C[160]QGPPGVDLYR	y6
	630.61	21.6	SLTEEAENWGDGEPNNK	y6
	631.82	25.17	TIAMDGTEGLVR	y8
	631.67	25.93	AHQGTGAGISPVILNSGEGK	y7
	631.84	27.04	LTC[160]VVESSVLR	y8
	630.67	27.2	VAVHPGGDTVAIGGVDGNVR	y6
GLAAGLDPNTATAGELAR	565.31	25.47	ELVAENLSVR	y8 y7
	564.82	24.8	ALGLASGELAAR	y6
	565.61	25.2	TVGEGEDHDIPIGIDK	y7
	566.63	25.64	VLETEAVDQPDVVQR	y6
	565.64	25.75	LIAPVAEEEATVPNNK	y7
	566.63	26.52	ILLTDGDPTVGETNPR	y6
	565.31	26.96	IEVLEEELR	y8
	565.95	27.53	VDVDIPDVNIEGPDAK	y6
	565.61	28.49	ALEPSAVQEEFMTSR	y7
	565.64	28.98	SRPVGHC[160]LEAAAVLSK	y8
	565.62	29.75	LRPIYDYLDNGNNK	y7
	565.35	29.83	IDISQLVITK	y8
	565.59	30.88	IFDLQDWTQEDER	y7
	566.3	31.3	DLPPDTTLLDLQNNK	y6
	565.28	31.53	AQATWTELPWPHEK	y8
	565.31	32.39	PSPIQEESIPIALSGR	y6
	565.61	32.98	AAGLAFSDGDQWTLSR	y7
	565.29	33.11	VDYLVTEEEINLTR	y6
	566.62	33.15	IC[160]DQISDAVLDAHLK	y7
	565.93	33.61	GFGFVTFDDHDPVDK	y7
	565.65	33.64	MSPPQLALNPSALLSR	y7
	566.28	33.86	C[160]GITVSQVSNWFGNK	y6
	565.28	33.93	ADMGGAATIC[160]SAIVSAAK	y8
TQIDQVESTAGSLQGQWR	668.31	26.79	FASENDLPEWK	y7 y6
	666.84	24.9	AGYAHFLNVQGR	y7

	668.32	25.37	SGFHGDC[160]LTLTK	y7
	668.88	25.71	IVEHPSDLIVSK	y5
	667.88	27.42	IHLPETVEQLR	y7
	668.37	27.56	LALYQTAIESAR	y7
	667.35	28.07	KPFDEIGGPAFR	y6
	668.04	30.22	LTGKPGQVLPHPELC[160]TVR	y7
	668.86	30.34	FVLDTSESVALR	y5
	667.34	30.37	ISHLGTPLYLAC[160]ENQQR	y5
	668.01	30.49	IPEISIQDMTAQVTSPSGK	y7
	668.68	30.68	EQANAVSEAVVSSVNTVATK	y7
	668.65	30.82	SDDIYNQVSAYPLPEHR	y6
	668.01	32.93	RPTEIC[160]ADPQFIIGGATR	y7
FLEGFVR	434.22	26.05	SNYLAWYQQK	y5 y4
	433.23	34.68	ATMC[160]NLLAYLK	y6 y5
	433.9	35.36	IFTMAEVYGIR	y5 y4
	434.55	25.98	SVTGGMC[160]SVYLK	y5
	432.89	26.24	TMFIGGSQLSQK	y5
	433.9	26.4	TSC[160]ALTIHAIGR	y4
	432.89	27.58	FTTDAIALAMSR	y6
	433.93	27.8	SLGVTQLAVAVNK	y5
	434.25	28.71	TVIVHGFTLGEK	y5
	433.25	28.9	DVQEIATVVVPK	y4
	433.54	29.2	VC[160]FGDFPTMPK	y6
	432.92	30.17	PPPSIPTFATLR	y5
	433.92	30.59	STLQTLPEIVAK	y5
	434.55	30.77	GAVYSMVEFNGK	y4
	433.26	32.05	LTLLHYDPVVK	y6
	434.22	32.39	DGTLVSFTADFK	y6
	433.9	32.63	SLIINTFYSNK	y5
	433.23	35.49	FMPLSDVLYGR	y5
AELPGVDPDK	520.77	18.64	HLC[160]AEAALR	y6 y3
	519.28	14.59	QHIQDGALR	y3
	519.78	16.62	VIVGGSSEYK	y7
	519.93	17.4	LQMEQQQQLQQR	y6
	519.6	17.83	SPVGSGAPQAAAPAPAAH	y6
	520.27	18.92	ETGYTELVK	y3
	520.57	18.96	HGIQYFNNNTQHS	y7
	519.6	19.48	ILSEVTPDQSKPEN	y3
	519.76	20.01	YSGTLNLDR	y6
	519.93	20.12	NGQIQYSIGGPNPGR	y6
	519.91	20.55	GASWIDTADGSANHR	y6
	520.59	21.14	LLETQEQEIEDGR	y3
ILVQANEAETTTASGLVIPDTAK	781.39	29.01	NEEQGIPLEYLEK	y5
	780.69	30.57	YPSDIAVEWESNGQPENNYK	y5
	780.91	31.48	IITDGVITDLDETR	y5
	781.42	31.92	MANITSSQILDQLK	y5
	779.9	32.49	SLPSLDEEPVTFPK	y5
	781.74	32.76	TETITGFQVDAVPANGQTPIQR	y9
	781.06	36.13	VFPPEVAVFEPSEAEISHTQK	y9
	781.38	36.59	ILSMANAGPNTNGSQFFIC[160]TAK	y9
	780.37	37.5	ISPDTSLLLDC[160]GEGTFGQLC[160]R	y9
	780.71	37.62	HPSAVTAC[160]NLDLENLITDSNR	y5
	779.92	38.48	C[160]DAVLC[160]TLPLGVLK	y10
	780.94	38.55	SLGLETAGGVMTALIK	y5
TADGINYR	453.74	11.35	GGGLSVSSSR	y5
	454.23	12.1	MGPPVGGHR	y5
	455.55	12.36	VSAGNENAC[160]LTTK	y5
	455.27	12.46	AAAVHTAIR	y5
	454.23	16.99	SEAIQTSHFQGR	y4
FLSAATSSTPR	568.24	16.76	ELVSC[160]SNC[160]TDYQAR	y8
	567.84	19.42	HLVLAGGSKPR	y6
	569.3	20.88	VAFTGSTEIGR	y7
	568.78	21.19	TQAFEADNLK	y6
	568.33	21.47	VALHNLINNK	y6
	567.81	23.84	ELGTGQFGVVK	y8
	568.31	23.84	QFVTATDVVR	y6
	568.25	23.84	ENYPNAGLTMNYC[160]R	y6
	569.28	24.72	YYYIPQYK	y6
	568.63	24.8	HHPTTLAVGIC[160]SPASR	y8
SLENYIAQTR	596.63	19.72	DSSSTDSANEKPALIPR	y8 y7 y6
	596.62	19.9	HGAGAEISTVNPEQYSK	y7 y6
	597.81	16.7	EAYGAVTQTVR	y8

	597.79	18.4	ESLSTAAEC[160]VK	y7
	596.83	21.22	VIDVPDAPAAPK	y7
	597.8	21.26	EAAENSLVAYK	y8
	597.34	23	ASAPLPGLSAPGR	y7
	597.61	23.26	QEC[160]SIPVC[160]GQDQVTVA	y6
	596.82	23.98	GALEAYVQSVR	y6
	596.29	24.28	AALADDFDTPR	y7
	596.8	24.76	AGQAVDDFIEK	y7
	597.64	24.76	SEAAHQGVITWNPPQR	y8
	597.93	25.11	PDGDVGGPWC[160]YTTNPR	y6
	597.3	25.43	FQEYHIQQNEALAAK	y6
	597.63	25.57	LVGAIVYYDGQHNDAR	y8
GLSTHEGALLSER	455.91	20.15	TQGLVPEHVETR	y4
	456.22	20.83	C[160]QNLYQLEGNK	y6
	456.59	21.99	LYRPGSVAYVSR	y4
	456.91	22.65	YGVSGSDQTLTIK	y4
	457.59	23.06	HTVGHIILSEHK	y5
	456.19	23.76	MC[160]PDYQSYFR	y4
	456.55	24.13	ALEQYEGESWR	y4
	456.73	24.2	HTGPGLLSM	y5
	455.9	24.97	GDFPSPIHVSGPR	y4
	457.55	25.72	LSC[160]C[160]QFAESLR	y4
	457.55	26.52	NTFTLSC[160]DGSLR	y4
	457.23	26.61	AEMDQILHGLDK	y6
	457.23	26.72	HIQPGAFDTLDR	y4
	456.58	26.75	FVQLEGAHPLEK	y5
	456.24	28.99	ITSGPFEPDLYK	y4
WETFLTSELPQWLSANR	1039.49	42.47	QQLVELVAEQADLEQTFNPSDPDC[160]VDR	y8
	1039.82	49.75	TQDHDENVALEAC[160]EFWLTLAEQPIC[160]K	y11
LYASAEATDSK	577.9	11.57	C[160]EQC[160]QPGYYGDAQR	y7
	577.61	13.31	DPPSEANSIQSANATTK	y8
	577.93	13.89	NQVIQTGPDEEGSDDK	y6
	577.81	14.05	THQGLSSPVTK	y8
	577.3	16.36	GTHHGLLANPH	y7
	578.28	16.45	GATVYATGTHAQVEDGR	y7
IESENPDAVANVQAR	N/A			
DGQLTIK	387.54	11.95	VTVSSASPTSPK	y4
	387.86	12.47	MENQALQEAK	y6
	386.72	12.68	AVVVC[160]PK	y5
	387.85	14	VDTC[160]HTPFGK	y4
	386.71	14.11	VANPLSTA	y5
	386.73	14.84	LEGIVNK	y6
	387.2	16.86	TVEVAEGEAVR	y4
	387.87	19.41	ITELTDENVK	y4
	386.54	19.65	IPASWTNPSGK	y4
SDGSGDTFLFTQYLSK	882.79	38.94	ASVPDGFLSELTQQLAQATGKPPQY	y9 y7
	881.91	32.78	YQLQSQENFEAFMK	y7
	883.77	35.25	DALPEYSTFDVNMKPVVQEPNQK	y7
	881.9	35.98	SLFTDLEAENDVLHC[160]	y7
	883.43	37.63	LGDVGMAELC[160]PGLLHPS	y7
	881.97	40.1	TFQTPDFIVPLTDLR	y9
	881.92	41.42	QDLEMYGINYFEIK	y9
	883.42	41.52	VEQIAAIAQELNELDYYDSHNVN	y9
VIAINAEPNGR	576.29	17.52	DVKPHNVMIDHEHR	y7
	577.3	19	AGYPTGTGVGPQAAAAAAK	y7
	576.27	19.81	LHHVSPADSGEYVC[160]R	y8
	576.58	21.76	SHSGNISC[160]MDFSSNGK	y4
	576.94	22.2	C[160]LGPEVTTQYGGQYR	y7
FLEGLTLR	473.9	26.32	LYDEMPPSALQR	y5 y4
	474.57	26.52	VDAETGDVFAIER	y6 y5
	474.57	27.26	DEELSC[160]TVVELK	y6
	474.28	27.32	YLGLPGSLK	y6
	473.58	27.6	GFSVVADTPELQR	y4
	474.91	27.72	QVAEAYEVLSDAK	y4
	474.61	27.78	VVSQHQALLGTIR	y5
	474.58	28.36	GQPLYLSC[160]ELNK	y5
	474.9	28.86	PASEVINEYSWK	y4
	474.3	29.28	IAFAITAIK	y6
	474.23	31.01	DLNMDC[160]IVAEIK	y5
	474.92	32.02	EHHFEAIALVEK	y5
	473.9	32.43	MAVQDAVDALMQK	y5
	474.25	32.44	INTQEYLDVLGR	y5

	473.6	33.26	ANTTAFLTPLEIK	y4
	473.61	34.78	LIQFLQASITER	y4
	473.93	34.9	VSGISFPTTELLR	y6
DSIYYVDANASIQEMLNVMEEHQVF	985.8	42.34	PGPASAGGSAEALLSDLHAFAGSAAWDDSAR	y10
	985.16	45.24	TNEIVEEQYTPQSLATLESVFQELGK	y10
	985.18	45.4	TPIVPVEDLPYTLSMVATIDSQHGIEK	y10
	984.03	45.94	IPYKPNYSLNLWSIMK	y10
	985.81	47.53	HSSSTVFDLVEEYENIC[160]GSQVNILSK	y9
	985.56	47.79	VPIPC[160]YLIALVVGALESR	y8
	985.54	47.79	NMTIPEDILGEIAVSIVR	y8
	985.47	50.02	DYEFMWNPHLGYILTC[160]PSNLGTGLR	y10
AAVEEGIVAGGGVTLLQAAPTLDEL	1260.58	38.15	LEQYEGPGFC[160]GPLASGTGGPFTTC[160]HAHVPPE	y12
	1261.18	41.97	LAQQYYLVYQEPIPTAQLVQR	y7
	1261.65	45.31	VHLC[160]AAQLLQLTNLEHDVYER	y7
	1261.09	46.51	VHNGIEYGDMQLIC[160]EAYHLMK	y7
LAATVADAVSTAR	622.33	27.53	IQLVEEELDR	y9 y8
	623.31	21.5	DLEDESTPIVK	y8
	623.59	21.95	MTC[160]VDVNEC[160]DELNNR	y8
	622.3	22.03	DIQMTQSPSSVSASVGDR	y8
	621.81	22.16	MHLAVVAC[160]GER	y8
	623.29	22.81	SPTASDMLHMR	y9
	622.3	22.85	DVQMTQSPSSLSASVGDR	y8
	622.64	22.98	ALQDLENAASGDAAVHQR	y7
	623.27	23.99	YYYAVVDC[160]DSPETASK	y7
	622.3	24.05	FYDVALDTGDK	y7
	622.29	26.62	SSGGSGGGVPEQEDSVLFR	y9
	622.64	26.7	ENADSLQASLRPHADEL	y7
	622.64	26.71	SPDGAHLTWEPPSVTSGK	y9
	623.28	26.89	SC[160]DNPYIPNGDYSPLR	y8
	622.28	27.08	SAPC[160]TVVQC[160]DLQEMAR	y9
	622.63	27.09	SHLLNC[160]C[160]PHDVLSGTR	y9
	623.28	27.16	DNFGGGNTAWEEENLSK	y7
	622	28.42	RPALNYPVYGETTQVR	y8
	623.36	28.47	PPLRPQQNPVLPVAGER	y7
	623.62	29.02	MSC[160]FSRPSMSPTPLDR	y8
ALGATPNTGPAPQGA	661.24	9.84	IEGDEEMHC[160]SD	y6
	660.97	12.81	GSSGTGGTATWKPGSSGPGSTGS	y10
	661.3	14.9	SPGQHDGTYFGR	y6
	661.88	17.09	GLAPQNKPELQK	y4
TVSLPVGADEDDIK	729.85	20.86	SGTHTLPVESGDMK	y8
	729.67	22.14	APEGTSAPGGGPGTLDDSATIC[160]R	y10
	728.37	22.57	LGHPDTLNQGEFK	y6
	728.35	22.58	AYSEVDGQVFQGR	y6
	728.73	22.59	RPGPAASPTPGPGPAPPAAPAPPR	y6
	728.39	22.83	ASAGPQPLLVQSC[160]K	y6
	729.29	23.37	DYEEVGVDSVEGEGEEEGEE	y8
	728.36	24.24	ITIYDQENFQGK	y6
	729.31	25.17	EEEC[160]HFYAGGQVYPGEASR	y8
	728.86	25.86	TPELNLDQFHDK	y10
	729.3	26.1	EDEEESLNEVGYDDIGGC[160]R	y6
	729.86	27.06	VEGDMQVPDLDIK	y10
	729.99	29.48	QPAIMPGQSYGLEDGSC[160]SYK	y10
VVAASELVVGDR	607.33	21.26	AAVGQEEIQLR	y7 y6
	607.33	29.03	SELQLSVSAGSPHPAIAR	y7 y6
	607.33	30.26	LTVIEPAWER	y7 y6
	607.26	30.54	FVEGVC[160]PFC[160]GYEEAR	y7 y6
	607.82	21.88	SPATLSLSPGER	y6
	607.96	21.94	VIESGPDQLNDNEYTK	y7
	607.61	22.95	PEC[160]HLFYNEQQEAR	y8
	607.28	23.54	NSVDELNNNVEAVSQTS	y6
	607.29	23.82	SGTLGHPGSLDETTYER	y7
	606.6	24.11	DESTDSGLSMSSYSVPR	y7
	606.61	24.79	DASFHC[160]AAHYSLPEGR	y6
	606.96	25.21	ATSYTITGLTENQEYK	y8
	607.62	25.29	NAQNNGSNFQLEEISR	y8
	607.97	26.15	GGSLLAGGGGFGGGSLSGGGGSR	y8
	606.61	26.33	VGSDMTSQQEFTSC[160]LK	y7
	606.65	26.63	NYLAWYQQKPGQPPK	y7
	606.63	26.68	TSMC[160]SIQSAPPEPATLK	y6
	607.29	26.78	NETLGGTC[160]LNVGC[160]IPSK	y8
	607.61	27.01	DNC[160]APESIEFPVSEAR	y6
	606.62	27.3	TVPIDDSSETLEPVC[160]R	y8

	606.97	28.02	ALVDHENVISC[160]PHLGAS	y8
	606.97	28.07	TQNC[160]FLNSEIHQVTK	y6
	607.3	28.37	MDRPNGLYPNYLNPR	y7
	606.86	29.11	QLLAGGIAGAVSR	y7
	606.84	29.79	LIINTFYSNK	y6
GAAGTAAQAAVVR	571.57	17.22	FC[160]SEAQFEC[160]QNHR	y7
	571.29	17.51	AVGEEVWHSK	y8
EALALALDQER	614.96	23.02	PGEEITYSC[160]KPGYVSR	y7 y5
	614.92	25.67	NIPGDFEC[160]EC[160]PEGYR	y6 y5
	614.84	22.1	IEGTPLETIQK	y6
	614.29	24.35	DLSINSTEC[160]LHVHC[160]R	y6
	614.3	24.71	LEQENDDLAHELVTSK	y7
	614.69	25.14	TPQPVTIKPPPPEVVSR	y5
	613.35	25.58	KPGPGEPLVFGK	y5
	614.82	25.67	TQMAEVLPSPR	y5
	614.33	25.7	HVVPAQVHVNGGALASER	y6
	614.3	26.35	NVLGHMQQGGSPTPFDR	y5
	613.61	27.1	TDDYLDQPC[160]LETVNR	y6
	613.94	27.23	FPAEDEFPDLSAHNNH	y5
	613.95	29.08	ISENMNLQFENQMNK	y5
	613.98	29.77	LQHVEDGVLSMQVASAR	y6
	613.95	30.09	ASMDVENPDYSEEILK	y7
	614.66	30.71	HHVLQTAHPSPLSVYR	y5
ASFLDQVHFQPLPPAVVK	664.98	36.42	MVSDINNAWGC[160]LEQVEK	y6
	664.33	36.8	QALVFQC[160]VGC[160]GAFHLQR	y6
	665.04	37.19	QLGTAYVSATTGAVATALGLK	y8
	664.34	37.21	AC[160]LDYPVTSVLPPASLC[160]K	y6
	663.35	37.37	SNVNFVTEIFR	y5
	663.69	37.5	SGPSGPDNVQSLPLWPLPK	y5
	663.9	37.98	LLGNVLVC[160]VLAR	y6
	664.01	38.02	WQVVDTPGILDHPLEDR	y5
	665.05	38.27	NVVVTQADQIGPLPSTLIK	y5
	663.88	38.39	VSIVNHLEFLR	y5
	663.35	38.96	EVAFWTNLFAK	y5
	665.34	39.46	NTFAEVTGLSPGVTYYFK	y8
	663.69	40.11	VQSSEMGTSVLIMQPILR	y8
	664.9	40.13	SNTIQSIIAIIR	y5
	664.88	40.14	VNIWLGLSAVEK	y5
	664.35	40.23	EDPANILQELPAPLLDDK	y6
	663.91	40.43	TTVILPLAPFVR	y5
	664.67	41.8	QETQLLEDYVEAIEGVR	y6
	664.35	44.47	LMATMFQNLFPSINVHK	y6
	665.03	45.11	ELFDVVANPLVNDLIHGK	y6
NYTAPGGGQFTLPGR	766.86	21.2	MADEAVC[160]VGPAPTSK	y11 y3
	767.39	28.02	SAGLEEIEQELTSK	y11 y3
	766.82	20.59	IDDPTDSKPEDWD	y3
	768.35	20.99	STAPETAIEC[160]TQAPAPASEDEK	y3
	767.98	21.57	C[160]LTTDEYDGHSTYPSHQYQ	y9
	768.37	21.76	NYTPQLSEAEVER	y9
	767.86	22.65	LEDALSSDTSGHFR	y9
	766.88	24.01	GSLISTDSGNSLPER	y9
	767.36	25.37	GWEEGVAQMSVGQR	y3
	767.86	25.87	YVIDYYDGGEVNK	y9
	767.11	26	KPVAAAAAAPAPAPAPAPAPAPAKPK	y11
	767.33	26.28	AFYEC[160]LAAC[160]EGSR	y11
	767.38	26.41	QVQEAIAGISNAAQATSPTDEAK	y11
	767.4	26.83	QLSQALDTSNVMVK	y3
	766.91	27.47	HADVGVALLANAPER	y3
	766.87	27.51	LLEGEDAHLSSSQF	y3
	768.32	28.45	C[160]EC[160]PVGFFYNDK	y11
	766.9	28.77	HSTFFPALQGAQTK	y11
	766.9	29.27	SDTLQITDLGVSGAR	y3
	767.38	29.52	EAQLDEEGQFLVR	y3
NDPLLNVGK	485.24	22.33	YHVVSGADDYTVK	y7 y6
	484.83	26.24	VLLLTGKPK	y6 y5
	484.72	17.85	MPEC[160]YIR	y7
	484.74	20.59	LPDGYEFK	y6
	484.59	20.96	GNVAYATSTGGIVNK	y5
	484.25	21.89	VPQFSFSR	y6
	484.22	23.2	HNC[160]AVEFNFGQK	y7
	485.6	24.88	APLHLHENPDLAK	y6
	484.59	25.04	NTFGSGVTSAVNVAK	y5

	483.92	25.4	KPPKPQLMANYY	y5
	485.26	25.94	TELISVSEVHPSR	y6
GGYFPVAPNDQYVDLR	905	38.37	GPHALGAPSLLLTGTQLY	y12 y7
	906.1	28.5	LVDEPGHC[160]ADFHPSGTVVAIGTHSGR	y7
	904.44	29.23	LIHGPNLYC[160]YSDVEK	y7
	904.97	29.41	GAAQLAELGPPGVGGVGAC[160]K	y7
	905.79	35.26	TPDTTANAEGDLPTTMGGPLPPHLALK	y7
	905.14	35.53	ATSTTELPPEYLTSPLSQQSQLPPK	y7
	906.11	36.61	GAELVEGC[160]DGILGDNFRPTQPLSDR	y12
	905.09	37.63	DSVHLTWEPPDDDGGSPLTGYVVEK	y7
EYAEVQPLK	537.28	15.99	IAQSAELADR	y7 y3
	537.28	17.81	TLVADVNADR	y7 y3
	537.78	15.35	TASPHFTVSK	y3
	537.28	16.53	EVQLVESGGR	y3
	538.28	17.09	ITC[160]SGEALPK	y3
	538.6	17.63	NQYVPYPHAPGSQR	y5
	538.81	20.14	TPASPVVHIR	y3
	537.3	20.18	VAEGQTLDLK	y3
	537.3	20.29	LTPTHYLTK	y3
	537.28	21.47	EVQLVESGGGV	y3
	538.78	21.92	YEYVLHPR	y7
	537.93	22.66	VASVMQEYTQSGGVR	y3
	538.79	23	MLQLVEESK	y3
	537.31	24.09	FLQVQPVSR	y3
	538.29	24.46	LLEQTQIIGEQNAR	y3
	538.62	24.58	IQDLSQQAQLAAAEK	y3
LVPGYEAPINLVYSQR	908.95	31.75	LLELTSSYSPDVSDYK	y10
	909.97	32.09	FYGPTNFSPIVNHVAR	y8
	909.77	33.77	SLSRPTTETQFSNMGMEDVPLATSK	y8
EATWLGDER	537.96	24.62	ALLQYADPVSAQHAK	y6 y5
	538.28	17.58	LAQFEPSQR	y5
	537.3	20.18	VAEGQTLDLK	y5
	537.58	20.23	FEFHYPNSDQAQK	y5
	538.79	20.99	LSC[160]LGASLQK	y7
	538.27	21.32	FSGSNSGNTATLTISR	y5
	537.8	23.44	AAMIVNQLSK	y5
	537.91	24.08	SFSDADLADGVSGGEGK	y7
	538.27	25.98	HGNQYIQVNEPWK	y5
SEIEEALR	472.77	19.9	LTLDIQNK	y6 y5
	473.25	22.49	HPLKPDNQPFPQ	y6 y4
	473.24	22.66	LAEQELIETSER	y5 y4
	472.76	14.49	IVDNLQSR	y5
	473.26	15.3	ANGTTVHVGIHPSK	y5
	473.25	15.6	SGDTPITVR	y4
	472.29	17.04	VPPPPPIAR	y4
	473.9	17.67	DAVC[160]SGVTGAANVAK	y5
	473.88	19.1	PEDVFTENPDEK	y4
	472.28	20.31	TVSPALISR	y4
	472.93	20.33	LAQAAQSSVATITR	y5
	472.9	20.5	FEAEPLPENTNR	y6
	473.24	21.18	SPTAPSVFSPTGNR	y5
	473.22	21.51	VDESDAFHDNLR	y5
	473.27	22.63	DIQTLLSR	y4
	472.28	23.43	VGADITVLR	y4
	473.24	23.67	PSSSPVIFAGGQDR	y5
	472.77	23.87	LEVDIDIK	y4
GSLVEGGIGGTEAR	650.65	18.3	EATPVVHETEPESGSQPR	y8 y7
	651.8	17.51	ALGADDSYYTAR	y8
	650.3	18.54	NPDGGFATYETK	y8
	651.31	18.82	SGENSAVLHYGHAGAPNDR	y7
	650.63	22	AGLGGAGASTSGSSGAC[160]GFSYK	y7
	650.66	22.34	FQPPQVPDQAPAEAPTEK	y7
	650.97	22.92	VHNPC[160]SDSVC[160]YSVAVTR	y7
	650.35	22.93	LVLAEAQVGDER	y7
	651.66	23.49	ASAEGPLLGPEAAPSGEGAGSK	y7
	651.33	24.04	EIEIDIEPTDK	y9
	650.84	24.53	LLQEALEAEER	y9
	651.64	25.19	FATDGEGYKPC[160]DPQVIR	y9
	651.83	25.47	NSIGDIIDHYR	y7
	651.63	26.3	TMHC[160]HLDAPANAISVC[160]R	y8
	650.62	26.42	EDVEATFPVHQPGNYSC[160]	y7
TQDVAVLQLR	571.98	27.76	STQAATQVVLNVPETR	y6 y5

	570.59	22.79	LAYNENDNTYYAMK	y7
	570.62	23.1	LHAVNAEEC[160]NVLQGR	y7
	571.94	23.89	FMETTDPSTASSLQAK	y5
	571.63	24.36	HLSPYATLTVGDSSHK	y5
	570.97	25.37	DQTKPTPLILDEQGR	y7
	571.95	25.68	VQPGHC[160]YHLYNGLR	y5
	570.65	25.85	APQAPLHSVVQQLHGK	y5
	570.96	26.24	SESAPTLHPYSPLSPK	y5
	570.3	26.81	LQSWLYSSR	y5
	571.81	26.83	NVLDGELLNR	y5
	571.61	27.15	AGPNTNGSQFFIC[160]TAK	y6
	571.93	27.64	LC[160]PSGMYTEYIHSR	y5
	571.31	27.71	IGPALSC[160]GNTVVVKPAE	y6
	571.35	27.77	ITNNINVLIK	y6
	571.26	27.85	DPETLVGYSMVGC[160]QR	y7
	571.61	28.31	AAHEALGQFC[160]C[160]ALHK	y5
	571.97	28.89	SAGIQVVGDDLTVTNPK	y5
	571.27	29.38	DNFVFGQSGAGNNWAK	y7
	571.28	29.45	GDVGQTVDDPYATFVK	y5
	571.76	29.67	VETNMAFSPF	y6
	571.3	29.81	VDIETPNLEGTLTGPR	y5
	570.61	29.86	NAYEESLEHLETFK	y7
	571.85	29.96	TLVGVGASLGLR	y7
	571.63	30.47	EAPC[160]VLIYIPDGHTK	y5
	571.31	30.65	TSGPNQEQVSPLTLLK	y5
	570.63	30.66	NSLGGFASTQDTTVALK	y7
	571.94	30.68	ESVNAAFEMTLTEGSK	y6
ATIEQLLTIPLAK	705.34	35.83	IFPHLC[160]SLC[160]NVEC[160]SHLK	y4
	705.05	36.1	QVQFHQGFGDLAVLKPSNK	y4
	705.35	36.36	VDVGGSEPASLSYLSFEGATK	y4
	704.89	37.27	EDTLLLIGHLER	y4
	704.69	37.29	GLELEPGAGLFVAQAGGADPDK	y7
	706.02	37.77	GAALDVHESEPFSFSQGPLK	y4
	705.02	38.06	IWTLEQPPDEAGSAAVC[160]LR	y8
	705.38	38.77	EAVGAFVVFDISR	y4
	705.36	39.19	SVTQGFSFNILC[160]VGETGIGK	y7
	705	39.42	SPPLIGSESAYESFLSADDK	y8
	706	40.24	LC[160]YGLNMDFVDPAQITMK	y7
	704.4	40.25	GLAFELVYSPAIK	y4
	705.74	40.42	IHQIELIPNDQLVAVISGR	y8
GLGEAQLGNSSGNFLLPDAQSIQA/	1030.16	44.07	GAVDDDVAEADIISTVEFNHSGELLATGDK	y12
	1030.2	44.87	HIPDEIFLLTAEQIAQEVSEQHLSQGR	y13
	1031.05	44.92	HEAGEALGAIGDPEVLEILK	y12
GISNFHSPSDVIVDASMPAMIR	782.05	39.98	AVSQVLDSLEEIHALTDC[160]SEK	y9 y5
	781.9	34.44	MMVC[160]QVGGIEALVR	y5
	780.41	34.45	TTLTGLDVQDMLPR	y5
	781.4	34.56	GNLASYPIDELVDR	y5
	781.92	35.35	TMPAFEVSLQALQK	y5
	781.72	36.51	GFDGIPDNVDAALALPAHSYSGR	y5
	781.4	36.74	EPQIEPTADLTGITNQLITC[160]K	y8
	780.71	37.62	HPSAVTAC[160]NLDLENLITDSNR	y5
	781.92	38.64	NSAASGLFIQAIQSR	y5
	781.38	38.69	VESVFETLVEDSAEEESTLTK	y9
	780.37	38.83	STQFEYAWC[160]LVR	y5
	781.43	39.23	AYIVQLQIEDLTR	y5
	780.47	39.73	AALSPLADLHALVLR	y5
	782.06	40.04	NIPPYFVALVPQEEELDDQK	y8
	781.37	40.49	C[160]ATQGTGLYEGLDWLSNELSK	y8
	781.4	41.09	ETVVEVPQVTWEDIGGLEDVK	y9
	781.45	42.52	AVSTLVEVLGGEFLK	y9
	781.39	42.73	VVQLSDAC[160]DMLGDHIVLDSVR	y9
	781.4	43.04	NLNPFEVLQIDPEVTDEEIK	y5
VQFQSGGANSPALYLLDGLR	702.36	35.46	C[160]PFAGILENGAVR	y10
	702.02	36.74	VHVASVNNFPTAAGLASSAAGY	y7
	702.33	37.93	MYFPDVEFDIK	y8
	702.87	37.96	PVEVMPVFPDFK	y7
	701.72	38.2	PC[160]PAPPVAGPSVFLFPPKPK	y10
	703.04	38.27	IGEHTPSALAIMENANVLAR	y10
	702.37	38.46	SWSPDVFSLPLR	y10
	702.04	39.41	GGTYMLNKPIEEIIVQNGK	y8
	701.69	39.73	GEEVAVPPGLVGYVMVTEEK	y7
	701.71	40.34	HSIELNEPPLVHTAASLFK	y10

	702.03	40.74	ELYERPPHLFAIADAAYK	y7
	702.01	40.96	FLSGSWSEFSTQPENFLK	y7
	702.01	41.4	EHAPSIIFMDEIDSIGSSR	y7
	702.99	41.64	GDVDMSEVEHFMPILMEK	y8
	702.68	41.78	IMGLDGATYDLEGHPQYLL	y7
	702.34	42.76	GVGVFGDMAQDTGIPADIWR	y8
	702.37	43.1	IMWNELETLVR	y10
IPDEDLAGLR	549.6	23.8	HSDFC[160]GPSPAPLHPK	y9 y8
	549.94	20.77	TAEC[160]TQYQQILHR	y7
	549.28	20.83	LDAGHTVYQVSQAEK	y9
	549.78	21.11	LHVDPENFK	y9
	548.94	21.45	VELHSTC[160]QTISVDR	y7
	548.97	22.49	VINQYQVVKPTAER	y7
	549.96	22.92	ATNGSGQATSTAELLVK	y7
	549.78	23.23	LDFVAPATGAH	y9
	548.6	24.07	LC[160]YVGYNIEQEQK	y8
	548.8	24.08	HGESVLVLDK	y7
	549.63	25.15	TGVAVNKPAEFTVDAK	y7
	549.83	25.21	PLTLAAVGAASK	y8
	549.83	25.84	ILAQDVAQLK	y9
	549.94	26.44	TLPSGC[160]FNTPSIEKP	y7
	548.95	26.47	PSPAQIMYNGQPITK	y8
	548.63	26.54	AFAGPSQKPETIELR	y9
	548.62	26.59	PAPTSEDLTSATNIVK	y7
	548.6	27.53	VNVEVEFAEPEPER	y8
	549.29	28.77	AVVEPYNSILTTHTT	y7
TFGFGFGR	443.91	25.98	LYVSNLGIGHTR	y6 y5
	443.9	22.88	LLLHYSPQAC[160]K	y6
	443.89	22.94	YVGVSSDSVGGFR	y5
	444.23	24.4	NQILNLTTDNAN	y5
	443.75	24.51	VDAAELLR	y7
	443.9	25.33	ADLSGVTEEAPLK	y7
	443.9	26.32	WALSQSNPSALR	y7
	444.6	27.43	VLHASLQSVLHK	y5
	444.54	27.7	YSFLHDSQTSF	y5
	444.91	28.95	APGQLALFSVSDK	y5
	443.91	30.67	PSPAISVSVSAPAF	y5
	443.54	30.71	YWEMQPATFR	y5
	443.56	31.11	HSNLMLEELDK	y5
	444.25	31.58	PAFEVSLQALQK	y7
	443.57	32.03	YAALYQPLFDK	y5
HPDSDIFLDDVTVSR	571.63	29.19	LAASIAPEIYGHEDVK	y6
	571.27	29.38	DNFVFGQSGAGNNWAK	y8
	572.63	29.62	HLNQGTDEDIYLLGK	y6
	571.31	30.57	GQVPENEANVVITTLK	y6
	572.64	30.7	LVVDDTC[160]TLVIPQSR	y8
	571.95	31.32	WLSTHDPNITWSTR	y6
	571.64	32.07	FTLTGLKPDTTYDIK	y6
	572.95	32.45	QYATLDVYNPFETR	y7
	571.65	32.83	DYIVALQHPVTTDIK	y6
	570.77	33.75	MADFWLTEK	y6
	571.27	33.94	ELDINTDGAVNFQEF	y7
	572.64	34.3	GLGLSYLSSHIANVER	y7
	572.62	34.31	YEAVGSVHQAWEAIR	y6
	571.61	34.45	GAGAFGYFEVTHDITK	y6
	571.29	35.08	ESAGLIPSPELQEWR	y6
	571.28	35.5	HVEDVPAFQALGSLND	y6
	572.29	35.89	FVDEGSLYGLSIATSR	y6
	571.3	35.95	WTAPEAINYGTFTIK	y6
	571.66	36.07	SHHLLGLEAVDRPLR	y6
	572.29	36.09	LYSNAYLNDLAGC[160]IK	y6
	571.31	36.91	LFVGGAPPEFQPSPLR	y6
	571.27	37.84	EFDFAAAYSYLDTAK	y7
	572.31	38.12	VIEDLSGPYIWVPAR	y8
FALNAANAR	473.25	16.5	LSENVIDR	y6 y5
	474.54	16.78	GEC[160]WC[160]VNPNTGK	y7 y5
	473.89	19.72	STSPESPYTHWK	y6 y5
	474.58	21.32	IIC[160]DNTGITTVSK	y6 y5
	474.24	12.85	SAVEDEGLK	y5
	473.76	13.52	VSGSELVQK	y6
	472.74	14.45	DNVAEQLR	y6
	473.92	15.55	GTTVTVSSASPTSPK	y6

	472.76	15.86	EIAENALGK	y5
	472.77	16	VSQQILEK	y5
	473.24	16.08	QVSVQPNFQQDK	y5
	472.76	17.48	GASALQLER	y7
	473.9	17.67	DAVC[160]SGVTGAANVAK	y5
	473.54	18.73	HYDGSYSTFGER	y5
	474.23	18.79	GEATVSFDDPPSAK	y7
	472.77	19.9	LTLDIQNK	y5
	473.88	20.12	AWSDDTSQLGPDK	y5
	472.91	20.58	VIDQEMQAIGGQK	y5
	473.23	20.75	GILSMANSGPNSNR	y5
	473.9	21.29	ETGTLESQLEANK	y5
	473.89	21.95	LTSDSTVYDYAGK	y5
	472.92	21.98	LAVANITNADSATR	y6
	473.25	22.49	HPLKPDNQPFPQ	y6
IPITGSNPK	463.55	18.31	ADEASELAC[160]PTPK	y7 y6
	462.75	13.01	HLAAVEER	y6
	463.22	13.38	MYGGHGSHVTSVR	y6
	462.55	15.7	PWC[160]HTTNSQVR	y6
	462.89	17.93	NPSDSAVHSPFTK	y7
	463.75	19.81	IDVHWTR	y8
	463.55	21.03	TTGIVMDSGNGVTH	y6
RIPLDVAEGDTVIYSK	591.63	36.4	TPPVLDSDGSFFLYSK	y7 y6
	590.82	29.16	LPEMEPLVPR	y6
	591.91	30.21	C[160]ELC[160]DDGYFGDPLGR	y5
	591.94	30.6	LAYYQAVDLDEC[160]ASR	y6
	591.62	30.66	TPAVEGLTEAEEEELR	y5
	592.6	31.04	DEISFHC[160]YDGYTLR	y5
	592.63	31.13	NLQNNAEWVYQGAIR	y6
	592.29	31.33	NNQFASFIDK	y5
	592.29	32.17	QHVVYGPWNLPQSSY	y6
	591.96	32.33	QDTYHYLPFSLPHR	y5
	592.64	32.97	LSDVTLVPVSC[160]SELEK	y7
	592.99	33.05	AGVVGPELHEQLLSAEK	y7
	592.67	33.34	IHFPVSVKPGEEVIPK	y6
	591.64	33.65	NLHVVFTMNPSSEGLK	y6
	592.97	33.77	MSSSDTPLGTVALLQEK	y5
	591.63	37.14	TTLVC[160]PYLVDTGMFR	y7
	592.01	37.78	HILGLPVGQHIYLSAR	y5
	591.32	38.01	NTLNPVWQTFSIPVR	y7
ITGNSSADDIATLAGSR	823.39	24.04	HGGTGFAGIDSSSPEVK	y11 y10
	824.88	23.76	QAETELC[160]AEAEELR	y9
	823.44	26.34	LQALQADYQALQQR	y9
	824.42	27.27	NRPFMGSISQQNIR	y9
	824.33	28.38	DGAPFC[160]PEC[160]YFER	y9
	824.06	29.86	LYHDPNVNGGTELEPVEGNPYR	y10
	823.46	30.18	SHVVSQHQALLGTIR	y9
	824.9	32.73	SPFEVNVGMALGDANK	y11
EAPYELNITSATYQSAIPPR	1109.53	29.84	TLAQAMLNNAQPDQYEAPDK	y11
	1110.57	34.14	YLTAQPAPTSEDLTSATNIVK	y11
	1109.83	34.36	STGSGNTYAYGVMDSGYRPNLSPEEAYDLGR	y13
VQFQSGGNNSPAVYLLDGLR	711.03	32.46	SDLQRPNPQSPFC[160]VASSLK	y7
	711.67	32.49	SAYLHPQQFDC[160]EPGVLGSK	y7
	712.63	33.03	YGGC[160]LGNMNNFETLEEC[160]K	y7
	712.36	35.79	DDIAQVDYVEPSQNTISLK	y10
	712.38	37.08	ILHLPTSWDWR	y8
	711.35	40.53	C[160]YELC[160]DTYPALLVVPYR	y7
YVLEELR	460.56	21.75	NSVASISTC[160]DGLR	y5 y4
	460.27	26.94	GQPLGPAGVQVSLR	y6 y4
	460.58	22.85	ITLVSSGSGTMGAAK	y4
	460.55	23.26	YVDGDLC[160]PDGIR	y4
	460.56	24.14	LQEGFGC[160]VVTNR	y4
	461.6	24.29	LLQQGLAQVEAGR	y5
	460.87	24.62	ADSGEGDFLAEGGGV	y4
	460.57	24.66	HNAEVAAFHLDR	y6
	460.58	25.14	PPILEGAVGGNEAR	y4
	461.22	25.57	YGVEQVGDMIDR	y4
	461.22	25.84	TANQLMNDFAEK	y5
	460.58	25.92	SPALTIENEHIR	y4
	461.54	26.45	DWHGVPGQVDAAM	y4
	459.74	26.47	DMPPAFIK	y5
	460.89	26.62	GMAAAGNYAWVNR	y4

	460.21	27.18	MLDVMQDHFSR	y4
	460.23	28.66	PSPEGMSEIYLR	y4
	460.76	29.7	GLFIIDDK	y5
	460.56	29.79	HSNFLGAYDSIR	y4
	460.27	29.84	NVVLQTLEGHLR	y4
	460.23	30.72	VC[160]C[160]EGMLIQLR	y4
	460.58	30.93	NTLYLQMNNLR	y4
TVGDVVAYIQK	596	23.46	ELTSTC[160]SPIISKPKPK	y7 y6
	595.98	25.28	VMLGETNPADSKPGTIR	y6 y5
	596.28	26.58	WSDQTYPEGTQAIYK	y7 y5
	596.29	28.39	DNPEATQQMNDLIIGK	y7 y5
	596.84	28.8	NMLLVGVHGPR	y6 y5
	596.31	29.54	ETTSTTWHIVSQAVAR	y6 y5
	595.96	29.97	GEPGPPDADGPLYLPYK	y7 y6
	596.28	20.24	EAAGEGPALYEDPPDQK	y7
	595.98	20.66	ISPQIQLSGQTEQTQK	y7
	595.94	20.95	GFVPAGESSSEAGGENYK	y7
	595.95	21.18	ETKPEPMEEDLPENK	y7
	595.96	21.32	APEAQVSVQPNFQQDK	y7
	596.6	21.41	C[160]GEEQGSDAALHFNPR	y6
	596.32	21.85	VEQQNTKPNIYDIPK	y7
	595.34	22.12	QLQLHLPANR	y7
	596.84	23.34	LEANHGLLVAR	y5
	596.8	23.38	DC[160]LTESNLIK	y7
	596.29	23.75	ATHYHLSSQVQEMAGK	y7
	595.32	25.16	MLSVHVVGYGK	y5
	596.97	25.32	TSIHEAMEQQSISISK	y6
	595.93	25.34	VFC[160]VEEEDSESSLQK	y7
	596.28	25.91	FASYGAHGAQFPDAYGK	y7
	596.29	26.04	STTSDIPHMLNQVESK	y7
	596.61	26.26	FYQEPNGETPSSLYR	y7
	596.81	26.35	ATAENEFVALK	y7
	596.29	26.48	DPGGITAGSTDEPPMLTK	y7
	595.61	26.6	EVNFQNGIEC[160]GGAYVK	y6
	596.81	26.62	TAAENEFVALK	y7
	595.61	27.21	STHSELLEDYYQSGR	y5
	595.82	27.67	MFIVNTNVPR	y7
	595.35	27.72	IVILEYQPSK	y5
	596.31	27.73	SVAGGFVYTYK	y7
	596.33	28.62	LLETHIHNQGLAAIEK	y7
	595.93	28.78	DLYEAIDSEGHSYMR	y7
	596.95	28.86	AEAAASALADADADLEER	y5
	596.3	29.99	NLGTALYYATQNC[160]LGK	y7
	596.94	30.02	C[160]IVTSTYGWTANMER	y6
	596.6	30.08	FLC[160]DEGAGISGDYIDR	y7
IALFGNHAPK	355.86	19.8	LEAEIATYR	y6
	356.5	21.37	DDNMFQIGK	y6
	355.53	23.06	LNSAIIYDR	y5
	356.18	23.5	MHAAFGGTFK	y4
	355.86	23.91	LLESYIDGR	y6
	356.85	24.64	NLASRPYTF	y4
	356.53	25.12	APYIAYLTR	y6
	356.51	25.74	YMIAEDLGR	y4
	356.18	26.94	LGC[160]GLLDYR	y5
DVLAVVSK	415.74	18.49	EVVAVSVAG	y5 y4
	415.19	15.3	TTFEHAHNMR	y6
	415.54	16.13	DALDQAQQVEK	y5
	414.53	16.4	SGMVQTEAQYK	y5
	414.25	17.39	LPNSVLGK	y5
	415.21	17.45	PASGEVEASQLR	y5
	415.89	17.67	APAPQPPSLPDR	y5
	415.25	18.14	GLNISAVR	y4
	415.55	18.74	AEAGQGDVSIGIK	y6
	415.21	19.24	PASEDLQDLQK	y5
	414.85	20.08	GGQHGGDWSWR	y4
	415.89	20.97	TEQSVALLEQK	y6
	414.88	21.12	EVDIGIPDATGR	y6
	415.23	21.49	LSQLEGVNVER	y6
	415.54	21.96	C[160]LEPEGAAELR	y6
	415.22	22.23	YHLTEPGLASR	y5
	415.55	22.25	GNSLPC[160]VLEQK	y6
	414.89	22.32	GQLEALQVDGGR	y5

	414.54	24.9	DFDPVHGDVIK	y4
AFDWDQAYR	585.64	32.58	VHQSVISSWLSTDPAK	y6 y5
	584.8	24.31	QYGNEVFLAK	y6
	585.27	25.67	C[160]QGSLQAEEYHLYR	y5
	585.83	25.85	SNDVLPVITGR	y6
	586.27	26.47	IDFFEDTER	y5
	585.93	26.64	SGSFSDADLADGVSGGEGK	y5
	584.96	26.91	SQDEAPGDPIQQLNLK	y7
	584.94	26.93	FAAKPHNPGFGMGGPMH	y5
	585.3	28.1	LSHEGPGSELPAGALYR	y5
	585.29	29.09	TPQMPPTTPSSSFFTK	y5
	585.33	29.51	LYQLLTQYK	y5
	586.31	30.54	HIVVSC[160]AAGVTISSVEK	y6
	585.98	31.62	KPIDYTVLDDVGHGVK	y5
	585.94	33.18	ALDVYC[160]PVQWEYGR	y6
ITQDLLDR	487.24	19.16	LASGVEGSDIPDDGK	y6 y4
	485.96	21.23	IITGKPSGAISGVQK	y6 y5
	486.6	24.96	AQQVSQGLDVLTAK	y6 y4
	486.25	17.7	QQPDTELEIQQK	y4
	487.25	18.45	EEDSANLKPSELK	y6
	486.9	19.7	PVEEYANC[160]HLAR	y5
	486.92	20.61	IEDEQALGSQLQK	y4
	487.28	20.93	LDESAIVVK	y4
	486.58	21.41	C[160]PAPGPHPALVEGR	y4
	487.57	21.7	TLTEPC[160]PLASESR	y6
	486.3	22.27	TQILVGVNK	y4
	486.59	23.33	PVVISQSEIGDASR	y5
	486.3	23.79	VIVVITDGR	y6
	486.57	23.88	PTSETVMYPAYAK	y4
	487.27	24.39	EQLSLLDR	y4
	486.92	24.56	QFVTPADVVSGNPK	y6
	486.23	25.19	NDLGHPFC[160]NNLR	y4
	487.57	25.29	HEAFETDFTVHK	y5
	486.28	25.8	SLAPSLLDR	y4
LEEENPEAAQALR	734.89	16.95	PFSAPKPQTSPSPK	y9
	735.67	21.17	IHQDSESGDELSSSSTEQIR	y9
	735.4	22.33	AADVEQLKPEEIK	y8
LYTSPEDFEK	613.63	16.88	AEPSAPGGGGSPGAC[160]PALGTK	y5
	613.83	21.03	ILTHVAEMQGK	y6
	613.97	21.05	GIGGGISYGQEGSSGAVSTR	y6
	614.94	21.38	TGIEQGSDAGYLC[160]ESQK	y6
	613.85	21.4	LQIAQLRPSSN	y5
	613.79	22.06	NAAGNFYINDK	y7
	614.82	22.63	YITASTFAQAR	y7
	613.3	22.77	GGTFYVEPAER	y7
	614.99	22.87	LGSRPQPAEAYAEAVQR	y5
	614.62	23.77	HNNC[160]MASHLTPAVYAR	y5
	614.68	24.56	LTTVC[160]PTVKPQTQGLAK	y6
	614.93	24.99	MWDPHNDPNAQGDAFK	y5
TTPSIVAFAR	531.91	20.89	FPSGNYPPC[160]IGDNR	y8 y7
	531.8	27.27	TVVEALFQR	y7 y5
	531.6	28.4	SAYALGGLGSGIC[160]PNR	y8 y5
	530.63	21.79	PASPTPVIVASHTANK	y7
	530.92	22.07	GNVEGATQSLAEQMR	y5
	531.77	22.27	LSVNSHFMK	y7
	530.91	24.23	IPIDNMTNEMEQR	y5
	531.23	25.49	AGYQDMPEYENFK	y7
	531.6	26.18	VGNLPHDIDENELK	y8
	530.93	26.29	PYQGPDAVPGALDYK	y7
	531.26	26.48	QLVDEFQASGGVGER	y8
	530.6	27.04	QEQIINTMTQDLR	y7
	531.93	27.79	INQLSEENGDLSFK	y5
	531.23	28.84	C[160]GFGGGAGSGFGGGYGGGL	y5
	531.27	29.27	AIC[160]TGTLQLALDC[160]R	y5
	531.94	30.55	GLGAFVIDSDHLGHR	y8
	531.62	30.68	QLPELPDVELPTNK	y5
AGANLFELENFVAR	774.72	39.74	AVPTGDMEDLPC[160]GLVLSSIGYK	y8
	775.42	43.4	QLEVINAIVDPSGSLDLLTGNR	y8
	774.74	45.12	TKPEEAC[160]SFILSADFPALVVK	y7
	775.43	45.28	VLGLQDTITAELVVIEDAAEPK	y7
VVFINTGFLDR	640.97	32.4	SGPPPSTVSEAEFEDIMK	y7 y6
	639.97	30.39	SSIGTGYDLSASTFSPDGR	y6

	639.85	30.89	LTLEDQATFIK	y7
	639.83	31.64	GEIAEAYADLVK	y6
	640.97	32.26	NPGYPQSEGLLGEC[160]MIR	y6
	640.31	32.4	TDGEGALSEPSATVTIEEL	y6
	639.68	32.45	VNPLGGAVALGHPLGC[160]TGAR	y6
	640.66	32.52	GEC[160]VPGEQEPEPILIPR	y6
	639.37	33.52	ATLGPAVRPLPW	y8
	639.68	33.65	PSPAISVSVSAPAFYAPQK	y7
	640.31	33.69	AGAEYVVESTGVFTTMEK	y7
	640.33	33.75	LLEETGIC[160]VVPGSGFGQR	y6
	640.34	36.01	MLDYEQAPNIQLSIGVK	y6
	640.63	36.26	FKPAYNPYTEPSMEVF	y7
	639.99	36.3	YPQLTGFHSDLHFLDK	y6
	640.66	36.38	FVLSGANIMC[160]PGLTSPGAK	y6
	639.35	37	ANLGVFSVFAPR	y8
	640.65	38.06	NGMAVAQFLESNPWVEK	y7
	640	38.17	IGAVPLIQGEYMIPC[160]EK	y7
	640.34	39.2	FLTAFLVQISAHSDQNK	y6
	640.02	39.44	EVELLKPIEDVTIYEK	y7
LQFTATTLSGAPFDGASLQGK	1055.5	32.66	AAFNAQNNGSNFQLEEISR	y10
	1055.03	33.86	AVGIDLGTTYSC[160]VGVFQHGK	y10
	1054.48	34.75	YDTYAYVGLTEGPSPGDFR	y12
	1054.84	34.76	TPSTMENDSSNLDPSQAPSLAQPLVFSNSK	y10
	1054.99	36.85	AMDIHFHSPAFQHPPTEF	y10
	1054.57	37.22	AEQTILPLVDEALQHTTTK	y10
	1054.01	37.79	LGQSLDC[160]NAEVYVVPWEK	y10
	1054.04	40.05	VVGPISGADLHGMLEMPDLR	y13
	1055.58	40.17	LALHVYQHGLTGFLGQVTR	y10
	1054.04	42.01	TC[160]YDLLRPDVVLETAWR	y10
GSVTPAVSQFNAR	666.36	18.68	HLGGSQQLLHNK	y7
	665.84	21.45	QGPDAVPGALDYK	y6
	666.96	21.46	NPC[160]HNGGLC[160]EEISQEVR	y9
	665.85	22.59	DPLLASGTDGVGTK	y7
	666.33	22.81	QIQVFEDEPAR	y9
	667.31	25.16	GSDFWVEAGHTK	y7
	666.32	25.16	QAHILLDC[160]GEDNVC[160]KPK	y9
	666	25.3	TRPTTLGSSQFSGSGIDER	y7
	666.98	26.6	ELGGLEGDPSPEEDEGIQK	y7
	666.97	26.97	DSATMSLDPEEEAEHPIK	y7
	667.62	27.43	DC[160]GFPDEGEYIVTAGQDK	y7
	666.3	27.7	YSFLHDSQTSF	y7
LVFLTGPK	437.55	22.82	C[160]IC[160]SQLSLTTK	y6
	437.21	22.84	AC[160]YPLGIC[160]AER	y6
	437.25	23.96	EWAGALVK	y5
	436.57	24.11	NGMVLKPHFHK	y5
	437.87	24.36	GC[160]SFLPDPYQK	y6
	437.87	24.52	FSDGDQWTLSR	y6
	437.9	25.87	GATWVVLGHSER	y5
	437.24	26.09	PNLPPETVDSLK	y6
	436.86	26.09	TATSEYQTFFN	y5
	437.76	27.77	LMGLEALK	y7
	437.76	28.33	IC[160]LDILK	y7
	437.56	30.9	IADFSDPAFISK	y6
VQFQGGGPHAVYLLDGLR	641.82	32.23	LC[160]PDISFFQR	y6
	643	33.05	GTIVLASPGWTTHSISDGK	y6
	643.31	33.64	TYVGPMTESLFPGYHTK	y7
	641.68	35.19	LAQHTLQALQSELDSLR	y6
	642.34	35.41	ILYHGYSLLYVQGNER	y7
	643	35.94	DLKPENILLDDNMQIR	y7
	642.02	36.35	LIILLTDGDPTVGETNPR	y7
	642.67	36.6	SNHSAQDSAVENLLLLSK	y6
	641.97	36.77	PENFSFPDDLQC[160]VDLK	y7
	642.35	36.86	GSNNLTALHPALFQNLSK	y7
	642.26	37.39	DNEVDFQEYC[160]VFLSC[160]	y8
	643.02	38.44	C[160]RPDQLTGLSLLPLSEK	y6
	641.98	39.18	DMLSELSTVMNEQITGR	y8
	642.66	40.48	DLVLSGDLGSLYAMTQDK	y7
	642.36	41.27	DIQAVATSLLPLTEANLR	y7
	642.33	41.95	NLSALENYNFELVDGVK	y7
TVTDQVGR	438.23	6.99	SLEAQAEK	y6
	438.21	11.18	C[160]SGPGLER	y4
	438.57	11.74	PAPTASSQAAVSAR	y5

	437.23	12.4	YAAVHVHTNAAR	y5
NDPTQQIPK	519.57	8.71	AGAPAEEGAEAADNQR	y6
	519.56	10.32	KPSTSDDSDSNFEK	y6
	519.92	14.86	EQLQTEQDAPAATR	y6
	520.55	15.93	MMNGGHYTYSENR	y6
DSFGEDYGVTIADGPMAGLLAR	1126.56	38.34	YYALVC[160]YGPGIPISTLHDGR	y8
	1127.56	40.28	GVNWAAFHPTMPLIVSGADDR	y8
	1128.23	43.31	GNPGFSGLNLNIPILSSLGSSAPSESHPSDFQR	y12
SVFDDGLAFDGSSIR	792.07	35.58	IESSSPTVVEGQTLDLNC[160]VVAR	y10 y9
	791.87	29.54	FAPYIDDLSEEQR	y10
	793.34	30.87	DVPNPNQDDDDDEGFSFNPLK	y8
	793.42	31.51	LQEEHSLQDVIFK	y9
	792.4	32.51	C[160]IYLVDSGGAYLPR	y9
	793.41	33.34	NVLC[160]GNIPPDLFAR	y9
	793.39	33.78	MSEPLQSVVDHMATHLGVSPSR	y8
	793.72	35.48	DYHMERPLLNQEHLEELGR	y8
	792.42	35.61	TPIQVESSPQPGLPAGEQLEGLK	y9
	793.39	35.63	FSALESQLQDTQELLQEENR	y8
	792.42	37.86	PNVHAAEFVPSFLR	y9
	791.9	38.38	GAMGIMLVYDITNGK	y10
SEFAYGSFVR	581.93	28.35	TEEIFYYYDTNTGK	y8 y7
	581.96	24.22	NYGILADATEQVGQHK	y7
	581.98	26.2	LNVFAKPEATEVSPNK	y7
	581.96	26.5	ATNHMGNVTFTIPANR	y6
	580.93	26.71	YSLGSSFGSGAGSSSFSR	y7
	581.93	27.22	SFFTASEGC[160]SNPLGGGR	y6
	581.8	28.12	LEEAVWRPY	y6
	581.96	28.45	PYPTFNPSSDVAALHK	y7
	581.96	28.92	LAGGYENVPTVDIHMK	y7
	580.33	29.83	VLISTDVWAR	y6
	581.28	29.9	QSVFFFSGDK	y8
	581.63	30.09	EWGSHAPTFQVQSIR	y6
	581.95	30.38	VTGQFLYQDSNWASK	y7
	581.63	30.96	TANEGGSLLYEQLGYK	y6
	581.63	31.4	GEDVPLTEQTVSQVLQ	y7
	581.95	31.41	AAFGLSEAGFNTAC[160]VTK	y7
	581.98	31.49	LIIEETKPC[160]VPVSMK	y7
	581.27	32.42	SAQEMFTYIC[160]NHIK	y6
	580.64	32.69	VIAPENLPPLTPYC[160]R	y6
	580.64	32.81	MSEALPLGAPDAGAALAGK	y7
	581.61	32.9	ENLMASDHLDTPMLR	y6
	581.96	33.35	EEVGEEAIVELVENGK	y7
	581.96	33.64	GDEEGVPAVVIDMSGLR	y6
VYQNAGGTHPTTTYK	545.27	10.55	GQGSSPVAMQK	y7 y5
	545.75	9.1	QDDHGYISR	y7
	544.76	10.91	LGPHAGDVEGH	y5
	546.58	14.29	QEAVVEEDYNENAK	y6
PGLPVEYLQVPSPSMGR	608.93	32.06	LEPEDFAVYYC[160]QQY	y7 y5
	608.98	37.03	HIIVAC[160]EGSPYVPVHF	y8 y7
	609.97	32.17	QLDEYSSSVANFLQAR	y5
	609.31	33.35	MVSISNYPLSAALTC[160]AK	y5
	608.84	33.56	DLSLLQIQMR	y5
	609.64	35.43	YNPLQDEWVLVSAHR	y7
	608.63	36.06	DPDNELYFAHGLFSAK	y8
	609.28	37	C[160]GGELVDTLQFVC[160]GDR	y7
	609.84	37.25	FQDQVLDLLK	y7
	608.29	37.49	FNEFMSNILT	y5
	607.86	37.57	GSLPITVTWLK	y5
	609.33	37.65	NLLYIYPQSLNFANR	y5
	609.32	38.01	FWYFVSQLK	y8
	610	38.1	IANILNSEELDIQDLK	y7
	609.35	39.15	SKPILDLGAEALIMQAR	y7
	608.97	39.61	LMQDDEMNIFINLTK	y5
	608.98	39.7	HVEDVPAFQALGSLNDL	y7
	609.97	40.45	FSQLAEAYEVLSDEVK	y5
	608.32	40.47	TGIVDISILTTGMSATSR	y7
	608.62	40.62	LDLMDAGTDAMDVLMGR	y7
	609.99	40.66	ALTLQDLDNIWAAQAGK	y7
	608.8	40.74	MMAFFDSLVR	y8
	608.95	41.08	SPYEPLNFHAMFQPF	y8
	609.01	41.79	LFYLALPPTVYEAVTK	y5
GVTEETTTGVLR	630.84	16.69	GFEKPSAIQQR	y6

630.65	18.95	NPFRPGDSEPPPAPGAQR	y6
630.89	19.49	DYHFEC[160]YHC[160]EDC[160]R	y8
631.29	20.22	TESPATAAETASEELDNR	y6
630.32	20.3	EMSGSPASGIPVK	y7
631.31	22.09	NRPPFGQGYTQPGPGYR	y6
631.82	23.36	DLTDAAIGPAYR	y7
631.82	25.17	TIAMDGTEGLVR	y8
