# Supplementary material for: Detection of Mycobacterium tuberculosis Peptides in the Exosomes of Patients with Active and Latent M. tuberculosis Infection Using MRM-MS
Source: PLoS One. 2014 Jul 31;9(7):e103811. doi: 10.1371/journal.pone.0103811 (PMC4117584; doi:10.1371/journal.pone.0103811)
Supplement: Methods S1 — Patients were sampled from the overall cohort of patients with unexplained cough for ≥2 weeks, based on the reference standard definitions for TB outcome classification described in the supplemental methods section. (PDF) [file pone.0103811.s006.pdf]

Supplemental Methods

Patients were sampled from the overall cohort of patients with unexplained cough for ≥ 2 weeks, based on the following reference standard definitions for TB outcome classification:

Confirmed Active TB Diagnosis Categories

All patients diagnosed as TB-positive fit into one of the following categories:

1. **Culture-positive pulmonary TB (PTB)**
   - ≥1 positive sputum or bronchoalveolar lavage (BAL) culture with >50 colony-forming units (CFU) on solid Lowenstein-Jensen media
2. **Culture-positive AND smear-or TB nucleic-acid amplification test (NAAT)-positive pulmonary TB**
   - Culture with <50 CFU but accompanied by either ≥1 positive direct Ziehl-Nielsen (DZN) or LED fluorescence (FM) smear microscopy, or a positive GeneXpert MTB/RIF results on sputum
3. **Not culture-positive but smear-positive and TB NAAT-positive pulmonary TB**
   - No positive cultures but ≥1 positive DZN or LED FM smear microscopy and a positive GeneXpert MTB/RIF result on sputum
4. **Extra-pulmonary TB**
   - Fulfills none of the criteria for pulmonary TB, but has ≥1 of the following:
     - Fine-needle lymph-node aspirate with positive AFB-smear examination
     - Cerebrospinal, pericardial, peritoneal, or pleural fluid with exudative fluid chemistries and/or lymphocyte-predominant cell differential

Confirmed Non-TB Diagnosis Categories

Fulfills none of the criteria for confirmed active TB above, and

1. **Not Active TB**
   - ≥2 negative *Mtb* cultures; no positive *Mtb* cultures, AFB smears, or GeneXpert results; and showed radiographic and/or clinical improvement by 2 months of follow-up without receiving TB therapy
     - i. **Latent TB Infection (LTBI) status:**
       1. LTBI-positive: Not TB, and a positive T-SPOT result by FDA criteria.
       2. LTBI-negative: Not TB, and a negative T-SPOT result by FDA criteria.

Unknown TB status

Fulfills none of the criteria for confirmed active TB or confirmed not TB categories; <2 negative *Mtb* cultures
